# Combined transcriptome and metabolome analysis revealed the molecular mechanisms of fruit skin coloration in pink strawberry

**DOI:** 10.3389/fpls.2024.1486892

**Published:** 2024-10-10

**Authors:** Wenfei Xiao, Aichun Liu, Wenguo Lai, Jianrong Wang, Xiaoyuan Li, Yan Zha, Bo Zhao, Xiaoyang Chen, Hong Yu

**Affiliations:** ^1^ Institute of Biotechnology Research, Hangzhou Academy of Agricultural Sciences, Hangzhou, China; ^2^ Seed Center, Zhejiang Provincial Seed Management Station, Hangzhou, China

**Keywords:** *Fragaria ananassa* duch., transcriptome, metabolome, anthocyanin biosynthesis, strawberry skin color

## Abstract

Elucidating the key genes and metabolites responsible for fruit skin color is essential for the breeding of strawberry varieties with beautiful fruit color. Here, transcriptome and metabolome analyses were used to identify the key genes and metabolites associated with fruit skin color in strawberry accessions of red skin (Kaorino), white skin (2012-W02), and the pink skin (Fenyu NO.1, the F1 hybrid of Kaorino and 2012-W02). The metabolomic data showed that the content of anthocyanin-related metabolites, such as p-Coumaroyl quinic acid, 5-Hydroxyconiferyl alcohol and Coumestrol were significantly higher in red-skinned strawberry line Kaorino than in the white-skinned line 2012-W02. The flavonoids and isoflavonoids such as syringetin and 2,7,4’-trihydroxy-isoflavone, were less expressed in the Kaorino than in the other two accessions. Transcriptome analysis revealed that the expression of genes involved in anthocyanin biosynthesis, such as *BZ1, F3H, CHS, CHI, DFR*, *4CL*, *PAL, CCR, 4CL, F5H, REF1* and *UGT72E*, were also significantly upregulated in the red-skinned line Kaorino compared to the white-skinned line 2012-W02, while the *HCT*, *CYP75B1*, *FG3*, *HIDH*, *IF7MAT*, *I2’H*, and *VR* was downregulated in Kaorino. Combined transcriptome and metabolome analyses revealed that the pathways of isoflavonoid biosynthesis and flavone and flavonol biosynthesis, and the phenylpropanoid biosynthesis pathway essential for anthocyanin synthesis were commonly enriched by DRMs and DEGs. In addition, the metabolites of peonidin 3-O-glucoside, 2’-hydroxydaidzein and daidzin, and the genes of *CYP93B2_16* and *UGT73C6* were detected and most accumulated in pink-skinned Fenyu NO.1. This result suggested that the main strategy for obtaining a red skin color is to enhance the upstream pathway of anthocyanin biosynthesis, including the phenylpropanoid biosynthesis pathway, and to restrict the downstream steps in the flavonoid biosynthesis pathway, such as the branch pathway of flavone and flavonol biosynthesis and isoflavonoid biosynthesis.

## 1 Introduction

Strawberry (*Fragaria × ananassa* Duch.) is a perennial herbaceous plant belonging to the genus *Fragaria* in the family Rosaceae. There are about 24 recognized *Fragaria* species in the world, which are mainly distributed in Asia, Europe and America (Staudt, 1962). China is the country with the wildest strawberry resources in the world, with 13 kinds of wild strawberry resources, accounting for about half of the world’s strawberry resources (Hou et al., 2018). This fruit has gained popularity in many countries because of its sweet and sour taste, unique flavors and high nutritional value, such as enriched vitamin C and vitamin A (Mezzetti, 2013). Due to the high economic, nutritional, and even health care value of strawberries, more than 2,000 varieties of strawberry cultivation have been explored through constant breeding by breeders, and cultivated strawberries are grown in almost every country in the world (Hardigan et al., 2021; Yuji et al., 1997).

Fruit quality of strawberry plants determines consumer demand. The color of the fruit skin is a critical aspect of both its appearance and nutritional value, and it plays a key role in determining the commercial and aesthetic value of the strawberry (Pomar et al., 2005; Lin et al., 2018). Research has shown that the main reasons why fruit skin vary in color are the levels and composition of anthocyanins and chlorophyll (Rosianskey et al., 2016; Zhao et al., 2022). Anthocyanins are responsible for red to purple pigmentation, and chlorophyll, composed of both chlorophyll a and b molecules, are responsible green pigmentation (Wang et al., 2020; Zhang et al., 2015). Skin coloration is a unique phase in the life cycle of strawberry plants and is mainly due to the accumulation of anthocyanin pigments. Anthocyanidin is a flavonoid compound that consists mainly of water-soluble plant secondary metabolites that accumulate in plant vacuoles in the form of glycosides (anthocyanins). Its composition and content determine the degree and type of redness of the fruit skin (Pascual-Teresa and Sanchez-Ballesta, 2008).

At present, the synthesis of regulatory mechanisms underlying anthocyanin production in strawberry has attracted considerable attention. Luo and Liu et al. identified 14 anthocyanins in strawberry plants by HPLC-MS and HPLC-DA and reported that cyanidin-3-glucoside, pelargonidin-3-glucoside, pelargonidin-3-rutinoside, and pelargonidin-3-(malonyl)-glucoside are the four major anthocyanins present in strawberry fruits (Liu et al., 2016; Luo et al., 2014). The total anthocyanin content varied among strawberry fruits with different fruit colors, and the major anthocyanin was pelargonidin-3-glucoside (Yuan et al., 2023). The metabolic pathway of anthocyanin has been clearly shown by previous studies (Hichri et al., 2011). The anthocyanin synthesis pathway begins with the phenylpropane metabolic pathway, in which cinnamic acid is converted to various types of anthocyanins through a cascade catalyzed by anthocyanin biosynthetic enzymes, including phenylalanine ammonia lyase (PAL), chalcone synthetase (CHS), chalcone isomerase (CHI), flavanone 3-hydroxylase (F3H), flavonoid 3-hydroxylase (F3’H), dihydroflavonoid reductase (DFR), anthocyanin synthetase (LDOX/ANS), and UDP-glucose-flavonoid glucosyltransferase (UFGT) (Almeida et al., 2007). Finally, glutathione S-transferase (GST) transports anthocyanin to plant vacuoles (Luo et al., 2018). In addition, fruit pigmentation in strawberry plants appears to be controlled by regulatory proteins called transcription factors (TFs), such as *MYB*, *bHLH*, *MADS*, and *WRKY* (Allan et al., 2008; Gonzalez et al., 2008).

Although these studies have improved our understanding of the molecular mechanisms underlying strawberry fruit color, still relatively little is known about strawberry fruit skin color. In this study, the combination of transcriptome and metabolome analysis was used to elucidate the changes in metabolites and gene expression in strawberry accessions with red (Kaorino), pink (Fenyu NO.1) and white (2012-W02) fruit skin. The key genes, metabolites, and metabolic pathways associated with skin color identified in this work will provide crucial information for understanding the mechanism of fruit skin coloration in strawberry.

## 2 Materials and methods

### 2.1 Plant materials and growth conditions

The red-skinned female parent is Kaorino originated from Japan (https://www.hinshu2.maff.go.jp/vips/cmm/apCMM112.aspx?TOUROKU_NO=19529&LANGUAGE=Japanese). The white-skinned male parent 2012-W02 is a selectively bred seedling derived from the Japanese strawberry variety IROHA-001 (https://www.hinshu2.maff.go.jp/vips/cmm/apCMM112.aspx?TOUROKU_NO=24429&LANGUAGE=Japanese). The pink-skinned accession Fenyu No.1 is the F1 offspring of Kaorino and 2012-W02. All strawberry plants were propagated by strawberry stolons. The stolon seedlings were planted in the greenhouse of Zhijiang Base of Hangzhou Academy of Agricultural Sciences in September 2022, and flowering occurred in mid-October 2022.

Fruit samples were collected from three different plants at 15 days after flowering (white fruit stage, S1), 20 days after flowering (turning stage, S2) and 28 days after flowering (ripening stage, S3). Three biological replicates were performed for each sample. The collected samples were then rapidly frozen in liquid nitrogen and stored at -80°C. These samples were prepared for subsequent metabolomic and transcriptomic analysis.

### 2.2 Metabolomic analysis

Metabolite profiling was performed using a widely untargeted metabolome method. The freeze-dried fruit skin from each sample was crushed into powder using a mixer mill (MM 400, Retsch). The lyophilized powder (100 mg) was weighed and extracted with 1.2 mL of 70% methanol. The samples were centrifuged at 12,000 rpm for 10 minutes. After filtering the supernatant, the filtrate was analyzed by UPLC-ESI-MS/MS system (UPLC: Nexera X2 system, Shimadzu, Kyoto, Japan; MS, 4500 Q TRAP, Applied Biosystems, Waltham, MA, USA).

Metabolites were identified according to the m/z values, secondary fragments, and isotopic peaks. Principal component analysis (PCA) was performed to classify and discriminate between samples using the R package pcaMethods. Orthogonal partial least-squares-discriminant analysis (OPLS-DA) (Galindo-Prieto et al., 2015) was performed using the R software package tools (Thevenot, 2016) to calculate the variable influence on projection (VIP) of metabolites. Metabolite levels between different comparison groups were analyzed using paired *t*-tests and their fold changes were calculated. The *p*-value of the *t*-tests was adjusted for false discovery rate (FDR). The differentially regulated metabolites (DRMs) were determined based on the thresholds of VIP>1, log2(fold change)>1 and *p ≤* 0.05. The DRMs were then mapped to the KEGG, HMDB and LipidMaps databases for functional and biological process annotation.

### 2.3 Transcriptome analysis

The fruit skins (1 cm wide and 0.2 cm thick along the fruit skin) in each sample were obtained. A total of twenty-seven samples (three plants, three fruit development stages and three replicates of each sample) were prepared for RNA extraction. Total RNA was extracted using a PureLink Plant RNA Kit (Invitrogen, Carlsbad, CA, USA), according to the manufacturer’s instructions. An Agilent 2100 Bioanalyzer was used to determine the quality of the RNA, after which the mRNA was purified using poly-T oligo. The library was constructed using the NEBNext Ultra RNA Library Pre Kit (NEB, USA). After quality assessment, the library preparations were sequenced on an Illumina HiSeq 2500 platform, and 150 bp paired-end reads were generated. Clean reads were obtained from the original data by removing reads with adapters, poly-N sequences, and low-quality reads. The clean reads were then mapped to the reference genome (Fragaria x ananassa Royal Royce Genome v1.0 Assembly & Annotation, https://www.rosaceae.org/Analysis/12335030) using HISAT2 software (Kim et al., 2015).

Gene expression levels were analyzed using the fragments per kilobase per million reads (FPKM) method and calculated using Cuffquant and Cuffnorm (v2.2.1) (Trapnell et al., 2010). DEGSeq2 was used to identify DEGs according to the criteria of a log 2-fold change>1 and q ≤ 0.01. Functional annotation of the transcripts was performed using the phyper package in R software. Gene function was annotated based on the following databases: Nr (NCBI non-redundant protein sequences); Nt (NCBI non-redundant nucleotide sequences); Pfam (Protein family); KOG/COG (Clusters of Orthologous Groups of proteins); Swiss-Prot (A manually annotated and reviewed protein sequence database); KO (KEGG Ortholog database); GO (Gene Ontology). Gene Ontology (GO) enrichment analysis of the differentially expressed genes (DEGs) was implemented using the GO seq R packages based on the Wallenius non-central hyper-geometric distribution (Young et al., 2010), which can adjust for gene length bias in DEGs. The KOBAS software (Mao et al., 2005) was used to test the statistical enrichment of DEGs in KEGG pathways.

### 2.4 Conjoint analysis of the transcriptome and metabolome

After obtaining the DEGs and DRMs, we selected the pathways commonly enriched in both DEGs and DRMs for further analysis. The corresponding transcripts and metabolites were filtered based on the annotation information from the KEGG database. Then, the corresponding transcripts and metabolites were mapped to the relevant KEGG pathways.

### 2.5 Quantitative real-time PCR validation

To evaluate the accuracy of the transcriptome data, we selected eight DEGs, *Fxa1Ag101086*, *Fxa7Cg103039*, *Fxa7Dg100111*, *Fxa7Dg102232*, *Fxa7Dg102859*, *Fxa4Cg202053*, *Fxa1Bg201911* and *Fxa1Cg102117* for expression validation. qRT-PCR was performed on a Roche-Light Cycler^®^ 96 system (Roche Diagnostics, Pleasanton, CA, USA). For each reverse transcription reaction, a total volume of 20 µL was prepared containing 10 µL of 2× qPCRBIO SyGreen Mix (PCR Biosystems, London, UK), 1 µL of each of the forward and reverse primers (10 pmoles) and 60 ng/µL of cDNA as template. qRT-PCR was carried out with denaturation at 95°C for 10 min and 45 cycles of amplification with denaturation at 95°C (20 s), annealing at 55°C (20 s) and elongation at 72°C (25 s). Three technical replicates of each of the three biological replicates were tested. The relative expression levels of each gene were analyzed via the 2^–ΔΔCt^ method (Livak and Schmittgen, 2001). *FaACTIN* was used as the reference gene for expression analysis (Amil-Ruiz et al., 2013). The primers were designed by using Primer Premier 5 (Premier Biosoft, CA, USA). All primers used for qRT-PCR are listed in **Supplementary Table S1**.

## 3 Results

### 3.1 Phenotypic variation and metabolite features

**Figure 1:**
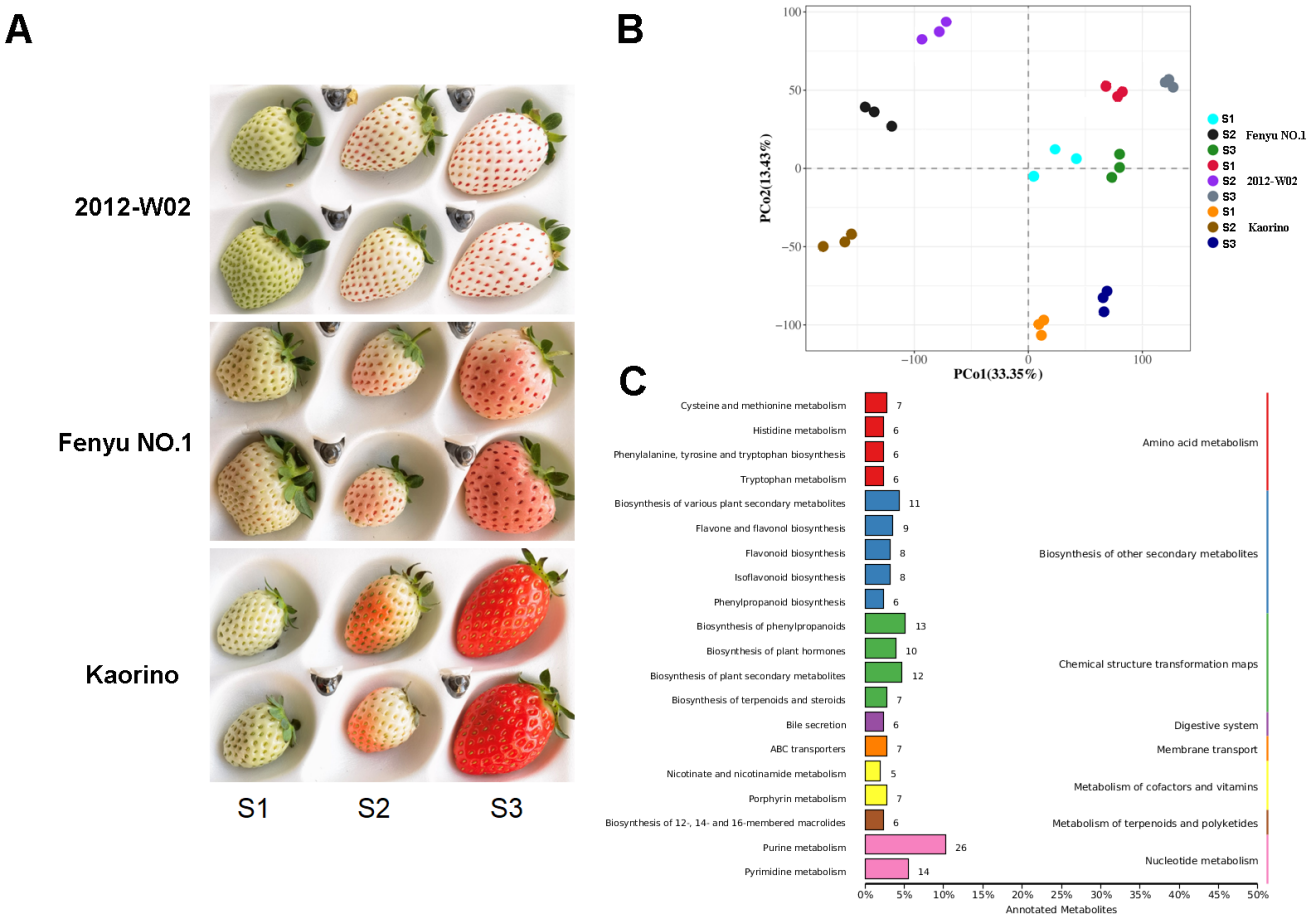
Phenotype and metabolite analysis of Kaorino, 2012-W02 and Fenyu NO.1. **(A)** Phenotypic comparison of fruit skin of three accessions (Kaorino, 2012-W02 and Fenyu NO.1) at three fruit development stages (S1, S2 and S3). **(B)** Principal component analysis of the samples. **(C)** Statistic of the detected metabolites annotated in different KEGG subcategories.

Distinct differences in fruit skin color were observed among the red skin accession Kaorino, the white skin accession 2012-W02, and the pink skin accession Fenyu NO.1 at three fruit development stages, especially at the S3 stage (**Figure 1A**). At the S1 stage, the 2012-W02 had light green skin color, while Kaorino and Fenyu NO.1 had white skin color. As the fruit developed, the skin color of 2012-W02 gradually changed to white by the S3 stage. In contrast, the skin color of the Fenyu NO.1 and Kaorino fruits changed from white to pink and finally to red. The skin color of the Kaorino fruits was significantly darker than that of Fenyu NO.1 fruits and appeared bright red at the S3 stage of fruit development (**Figure 1A**).

**Figure 2:**
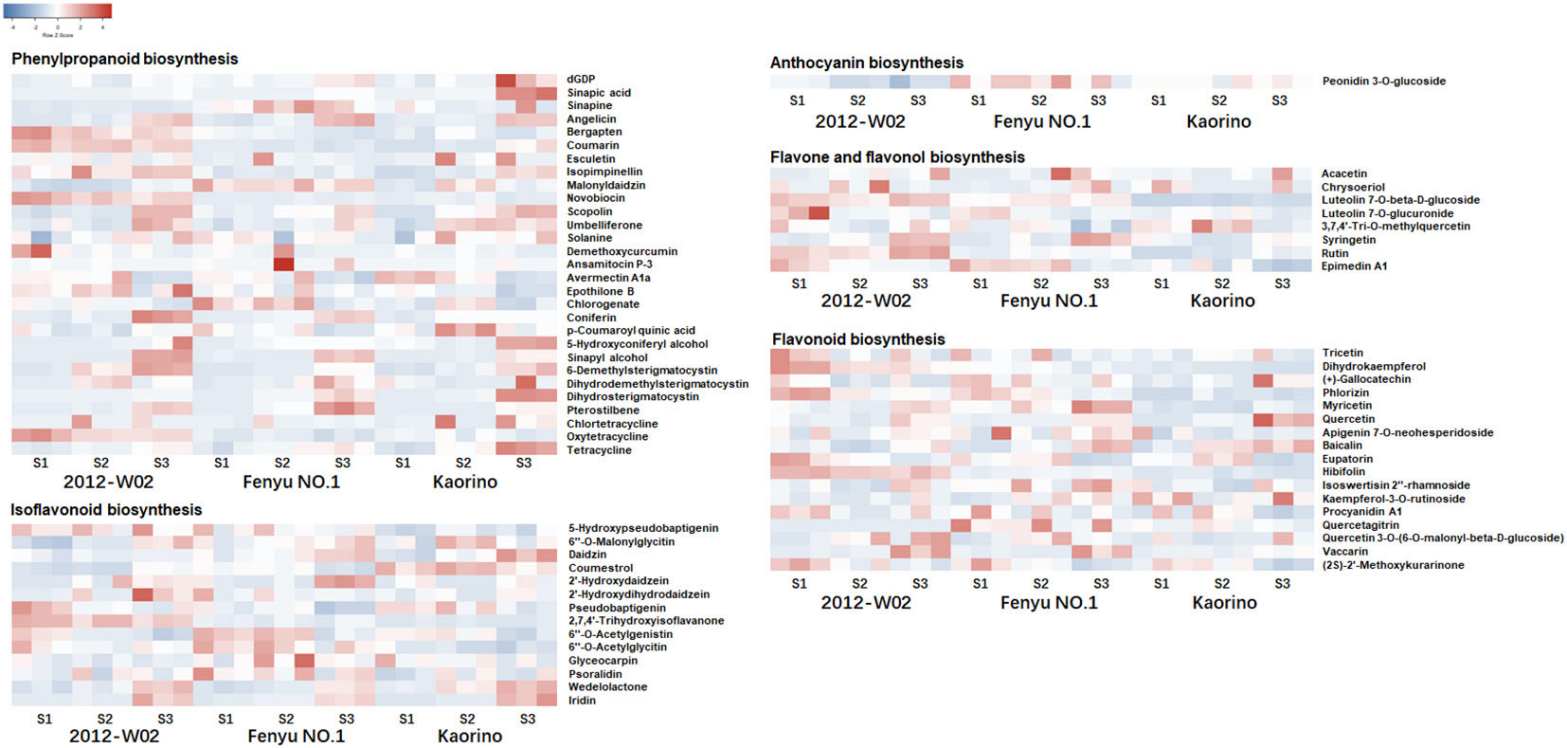
The accumulation of anthocyanin related metabolites in Kaorino, 2012-W02 and Fenyu NO.1 at stages of S1, S2 and S3. The red color means high accumulated, and the blue means low accumulated of metabolites. Each column represents a sample; each sample has three replicates; each row represents a metabolite.

To further investigate the differences in metabolite accumulation among these three accessions, metabolomic profiles were obtained for each accession at the three different developmental stages. A total of 1,057 metabolites were obtained using a metabolome detection system (**Supplementary Dataset S1**). The PCA analysis revealed a clear trend of isolation among the three accessions at different developmental stages based on the detected metabolite variables (**Figure 1B**). The first principal component (PCo1, 33.35%) effectively distinguished between the S1, S2 and S3 stages, and the second principal component (PCo2, 13.43%) separated the 2012-W02, Kaorino and Fenyu NO.1 accessions (**Figure 1B**). According to the enrichment of these metabolites in the KEGG database, the top 20 most enriched pathways are shown in **Figure 1C**. The majority of the identified metabolites were found to be involved in the pathway of purine metabolism, followed by the pathways of pyrimidine metabolism, biosynthesis of phenylpropanoids, biosynthesis of plant secondary metabolites, biosynthesis of various plant secondary metabolites and biosynthesis of plant hormones (**Figure 1C**). In addition, HMDB and LipidMaps annotations were performed for these metabolites (**Supplementary Figure S1**), and the anthocyanin-related metabolites were then identified by integrating the results of KEGG, HMDB, and LipidMaps annotations. A total of 69 anthocyanin-related metabolites were identified, including one metabolite involved in anthocyanin biosynthesis, 8 metabolites involved in flavone and flavonol biosynthesis, 17 metabolites involved in flavonoid biosynthesis, 29 metabolites involved in phenylpropanoid biosynthesis, and 14 metabolites involved in isoflavonoid biosynthesis (**Figure 2**; **Supplementary Dataset S2**). All these anthocyanin-related metabolites accumulate differently in three cultivars at different growth stages. For these phenylpropanoid biosynthesis related metabolites, half of the metabolites were accumulated more at S3 stage in three accessions (**Figure 2**). More p-Coumaroyl quinic acid was found in red skin line Kaorino than in white skin line 2012-W02 at S2 stage, and the same pathway related metabolite, 5-Hydroxyconiferyl alcohol, was also more accumulated in red skin line Kaorino than the other two lines at S1 stage (**Figure 2**). Likewise, the other anthocyanin-related metabolites involved in flavonoid biosynthesis, flavone and flavonol biosynthesis and isoflavonoid biosynthesis also showed the more accumulated at S3 stage in three accessions. At the S2 developmental stage, the metabolite related to isoflavonoid biosynthesis, Coumestrol, was most abundant in red skin line Kaorino and least abundant in white skin line 2012-W02. Moreover, the metabolites, Peonidin 3-O-glucoside directly participated the pathway of anthocyanin biosynthesis was detected and most accumulated in pink skin line Fenyu NO.2.

### 3.2 Differentially regulated metabolite analysis and annotation

**Figure 3:**
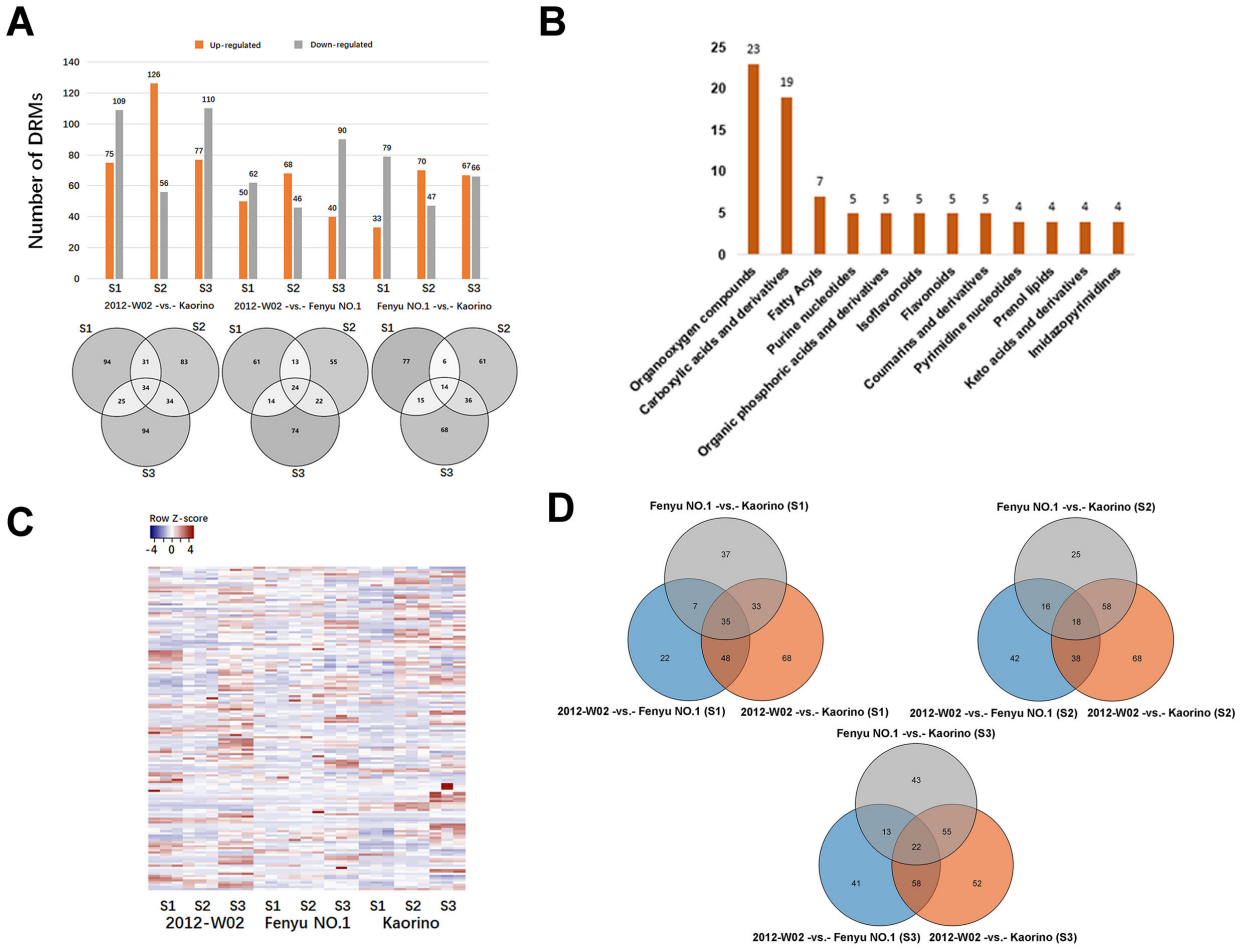
Analysis of differentially regulated metabolites (DRMs). **(A)** The statistic of DRMs number. The bar diagram reflected the number of upregulated and downregulated DRMs in each comparison groups, and the Veen diagram showed the number of DRMs identified in pairwise comparison of three samples at three development stages. **(B)** The categories of DRMs classified in HDMB database. All DRMs classified to 42 categories according to HDMB taxonomy, there showed the top twelve categories of most DRMs annotated. **(C)** The expression pattern clustering analysis of DRMs in three accessions. Each column represents a sample; each sample has three replicates; each row represents a metabolite. **(D)** The Venn diagram showed the number of DRMs at three compared groups at same development stages.

A total of 514 DRMs were filtered from the 1,057 metabolites with a threshold of VIP>1, log 2-fold change>1 and *p ≤* 0.05. There were 34 DRMs in the 2012-W02 -vs.- Kaorino comparison group that were detected in three stages simultaneously and the number of DRMs in S1, S2, and S3 were 184, 182, and 187 respectively (**Figure 3A**). The 15 out of 34 DRMs were more accumulated in Kaorino (**Supplementary Dataset S3**). For the 2012-W02 -vs.- Fenyu NO.1 comparison group, a total of 112, 114, and 134 DRMs were detected in three stages, respectively (**Figure 3A**). The 24 DRMs commonly present in three stages including 9 DRMs up-regulated in Fenyu NO.1 (**Supplementary Dataset S3**). In addition, a total of 112, 117, and 133 DRMs were identified in the Fenyu NO.1 -vs.- Kaorino comparison group at the three stages, respectively, and 14 DRMs were commonly present at the stages of S1, S2, S3 (**Figure 3A**). Most of these 14 DRMs were more abundant in Fenyu NO.1 than in Kaorino (**Supplementary Dataset S3**). More DRMs were identified in the 2012-W02-vs.- Kaorino comparison group than in the other two comparison groups (**Figure 3A**). In the three comparison groups, more DRMs were identified in the S3 stage than in the S1 and S2 stages (**Figure 3A**).

The DRMs annotated in both the KEGG and HMDB databases were filtered, resulting in a total of 151 DRMs obtained. Based on the HMDB annotation results, the top ten categories of these 151 DRMs included organooxygen compounds, carboxylic acids and derivatives, fatty acylspyrimidine nucleotides and prenol lipids (**Figure 3B**). The changes in DRM contents were significantly influenced by the different fruit skin colors (**Figure 3C**; **Supplementary Dataset S4**). The DRMs belonging to flavonoids (syringetin, baicalin, isoswertisin 2’’-rhamnoside, chrysoeriol and luteolin 7-O-beta-D-glucoside) showed higher accumulation levels in white skin accession 2012-W02 and pink skin accession Fenyu NO.1 compared to red skin accession Kaorino (**Table 1**; **Figure 2**). The levels of two DRMs belonging to isoflavonoids, wedelolactone and daidzin, gradually increase with the depening of skin color, with the lowest levels found in the white skin accession 2012-W02 and highest in the red skin accession Kaorino (**Table 1**).

**Table 1:** The regulation of DRMs involved in anthocyanin biosynthesis in each compared group.

Compound classification	DRMs	2012-W02 -vs.- Kaorino (Log2FC)			2012-W02 -vs.- Fenyu NO.1 (Log2FC)			Fenyu NO.1 -vs.- Kaorino (Log2FC)		
		S1	S2	S3	S1	S2	S3	S1	S2	S3
Isoflavonoids	Coumestrol	–	1.54	–	–	–	-0.67	–	0.73	0.95
	2,7,4’-Trihydroxyisoflavanone	-3.61		-3.02	-2.62	-2.46	-1.82	–	–	–
	2’-Hydroxydaidzein	–	–	–	–	–	1.1	–	–	-1.86
	Wedelolactone	–	–	–	–	–	–	–	0.7	
	Daidzin	–	–	–	–	–	0.71	–	–	–
Flavonoids	Syringetin	-2.41		–	-0.61	–	-0.48	-1.81	–	–
	Baicalin	-1.41	-1.71	-2.02	-1.2	-0.81	-0.88	–	-1.75	-1.54
	Isoswertisin 2’’-rhamnoside	–	–	-1.41	–	–	–	–	–	-0.53
	Chrysoeriol	-1.63	–	-0.7	-0.95	–	-1.08	–	–	–
	Luteolin 7-O-beta-D-glucoside	–	–	-1.20	–	–	–	–	-0.59	–

- means no significant difference. The value under each compared group is the log2 FC. The negative value indicated that this metabolite is down regulated at this compared group, while the positive value indicated up regulated.

The KEGG pathway enrichment for DRMs was further conducted to investigate the key pathways involved in skin coloration. The results showed that the DRMs were significantly enriched in glyoxylate and dicarboxylate metabolism, amino acid metabolism, biosynthesis of phenylpropanoids, flavone and flavonol biosynthesis and isoflavonoid biosynthesis (**Supplementary Figure S2**). Overlap analysis of the DRMs from the nine compared groups revealed that 35, 18 and 22 DRMs overlapped at the S1, S2 and S3 developmental stages, respectively (**Figure 3D**). For the comparison groups of 2012-W02 -vs.- Kaorino and 2012-W02 -vs.- Fenyu NO.1, there were 12 DRMs that occurred commonly at three stages (**Supplementary Dataset S5**). Among these commonly identified DRMs, the content of syringetin decreased as the skin color deepened, with the highest in the white skin accession 2012-W02 and the lowest in the red skin accession Kaorino (**Table 1**). In addition, the DRMs of 2,7,4’-trihydroxyisoflavanone were also commonly identified at three developmental stages at the compared group of 2012-W02 -vs.- Fenyu NO.1, and they were downregulated in the Fenyu NO.1 (**Table 1**).

### 3.3 Transcriptome statistic

Transcriptome sequencing of the skins of the three accessions at different develop stages yied a range of 38,297,972 to 53,015,656 clean reads, with Q30 values ranging from 93.13% to 94.47% (**Supplementary Table S2**). The mapping results showed that the number of mapped reads ranged from 36,360,914 to 50,224,230, accounting for 92.65% to 95.66% of the total number of clean reads (**Supplementary Table S2**). This indicated that the transcriptome data were of high quality and suitable for further analysis.

### 3.4 Identification and functional enrichment of DEGs

**Figure 4:**
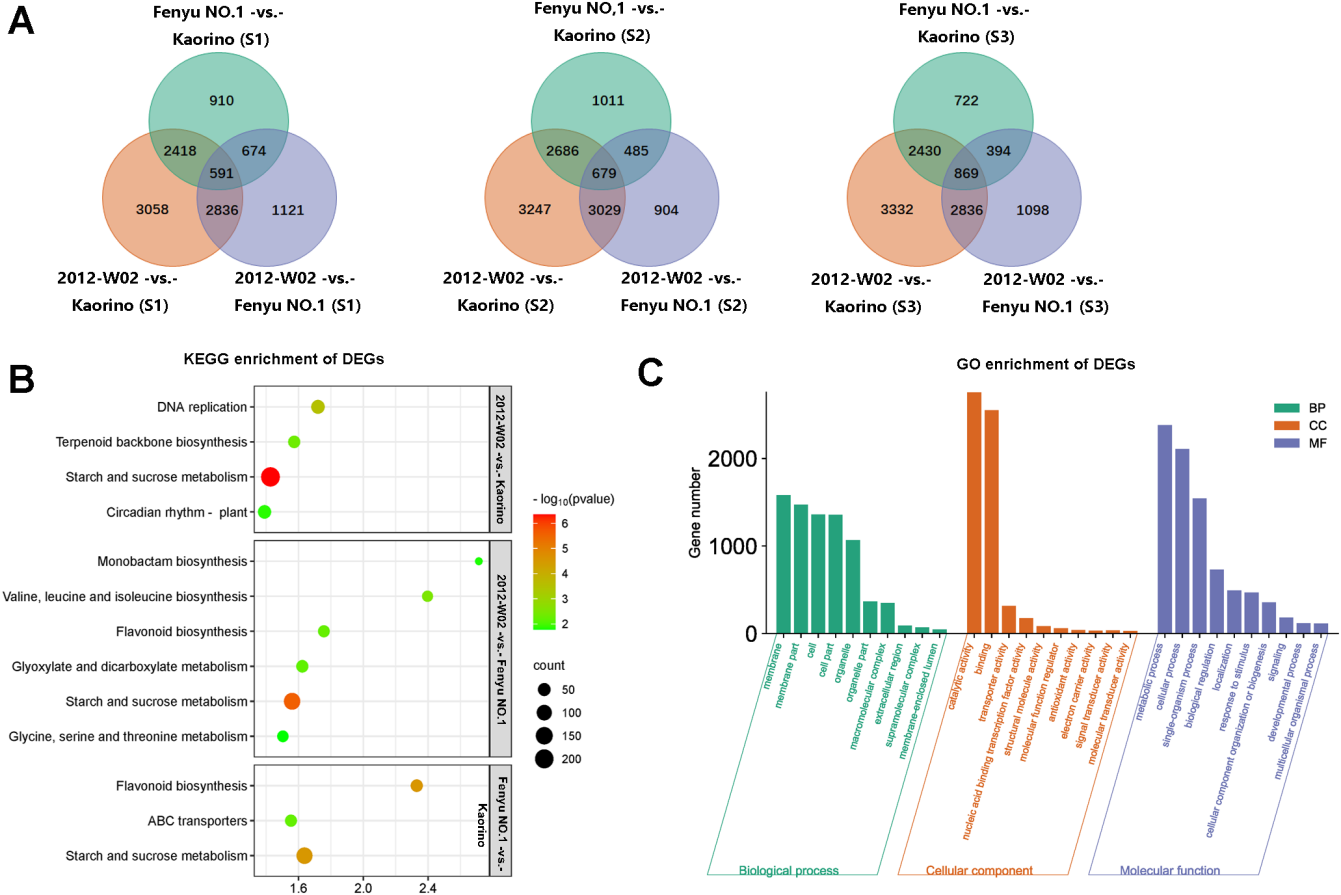
The functional annotation of DEGs. **(A)** The Venn diagram showed the number of DEGs at three compared groups at same development stages. **(B)** The top five enriched KEGG pathway of DEGs at compared groups of 2012-W02-vs.- Kaorino, 2012-W02-vs.- Fenyu NO.1 and Fenyu NO.1 -vs.- Kaorino. Bubble diagram obtained by selecting the top five significantly enriched pathways according to the number of DEGs. **(C)** The GO enrichment of the DEGs. According to the GO annotation of all DEGs in each compared group, we selected most significantly and most DEG enriched GO terms in each compared group, and finally obtained the top 10 GO terms at three GO classification of biological process, cellular component and molecular function. Then obtain the histogram of GO enrichment.

A total of 19,918 DEGs were identified among these nine compared groups (2012-W02-vs.- Kaorino, 2012-W02-vs.- Fenyu NO.1 and Fenyu NO.1 -vs.- Kaorino at S1, S2 and S3 development stage, respectively) (**Table 2**). At three developmental stages, the number of DEGs including up-regulated and down-regulated DEGs was shown in **Table 2**. The results showed that there were more DEGs in the 2012-W02-vs.- Kaorino (number of DEG at three development stage: 8,903, 9,641 and 10,578) than in the 2012-W02-vs.- Fenyu NO.1 (number of DEGs at three development stage: 5,222, 5,097 and 6,308) and Fenyu NO.1 -vs.- Kaorino (number of DEGs at three development stage: 4593, 4,859 and 4,415), and the number of DEGs at the S2 and S3 stages was greater than that at the S1 stage in the three compared groups. The 591 DEGs were detected spontaneously in three comparison groups at S1 stage, while the 679 and 869 DEGs were detected at S1 and S2 respectively (**Figure 4A**). In addition, two comparison groups, 2012-W02-vs.- Kaorino and 2012-W02-vs.- Fenyu NO.1, had more co-occurring DEGs at each stage than the other two comparison groups (**Figure 4A**). A total of 1,294 DEGs were detected to co-occur at three stages in the 2012-W02-vs.- Kaorino and 2012-W02-vs.- Fenyu NO.1 comparison groups (**Supplementary Dataset S6**). These 1,294 DEGs were identified between white skin accession 2012-W02 and red and pink skin accession Kaorino, Fenyu NO.1, and we believe that these genes are closely related to the development of strawberry fruit skin color.

**Table 2:** Statistics of differentially expressed genes (DEGs) in each compared group.

Stages	Regulated	Compared groups			Total
		2012-W02 -vs.- Kaorino	2012-W02 -vs.- Fenyu NO.1	Fenyu NO.1 -vs.- Kaorino	
S1	Down	5,007	3,278	2,472	19,918
	Up	3,896	1,944	2,121	
S2	Down	5,288	2,881	2,397	
	Up	4,353	2,216	2,464	
S3	Down	6,401	3,852	1,895	
	Up	4,177	2,456	2,520	

To elucidate the biological functions of these DEGs, GO and KEGG enrichment analyses were conducted. The plant pathways of starch and sucrose metabolism, DNA replication, terpenoid backbone biosynthesis and circadian rhythm were significantly enriched among the DEGs in the W02-vs.- Kaorino comparison group at all three stages (**Figure 4B**). For the comparison groups of the Fenyu NO.1 -vs.- Kaorino, starch and sucrose metabolism, flavonoid biosynthesis and ABC transporters were significantly enriched in DEGs at all three stages (**Figure 4B**). In addition to the starch and sucrose metabolism pathway, the pathways of flavonoid biosynthesis; glyoxylate and dicarboxylate metabolism; valine, leucine and isoleucine biosynthesis; monobactam biosynthesis; as well as glycine, serine and threonine metabolism were consistently enriched in DEGs at three stages in the 2012-W02-vs.- Fenyu NO.1 comparison group (**Figure 4B**). GO enrichment analysis of the DEGs in each comparison group revealed that membrane and membrane part were the most significantly enriched terms in the cell component subcategory. Catalytic activity, binding, and transporter activity were the most enriched terms by DEGs in the molecular function subcategory, while metabolic process was the most enriched term in the biological processes subcategory (**Figure 4C**).

### 3.5 DEGs involved in the fruit skin color of strawberry plants

To identify the genes related to the fruit skin color in strawberry plants, the DEGs commonly identified in the Fenyu NO.1-vs.- Kaorino, 2012-W02-vs.- Fenyu NO.1 and 2012-W02-vs.- Kaorino comparison groups at the three fruit development stages were analyzed. A total of 19 DEGs were identified and shown to be involved in pathways related to anthocyanin biosynthesis (**Table 3**; **Supplementary Dataset S7**). Among them, the expression levels of two anthocyanidin 3-O-glucosyltransferase (BZ1)-encoding genes, *Fxa7Dg102859* and *Fxa7Ag203300*, which are involved in anthocyanin biosynthesis (ko00942), were found to be highest in red skin accession Kaorino at all three developmental stages compared to the other two accessions. Conversely, these genes showed the lowest expression levels in the white skin accession 2012-W02 (**Table 3**; **Supplementary Dataset S7**). Similar to the expression tendencies of these two genes, the genes enriched in the Flavonoid biosynthesis pathway (ko00941), including the four flavanone 3-hydroxylase (F3H)-encoding genes (*Fxa1Bg201013*, *Fxa1Ag101086*, *Fxa1Cg101029*, *Fxa1Dg200969*), four chalcone synthase-encoding genes (*Fxa7Ag200133*, *Fxa7Bg200132*, *Fxa7Cg100092*, *Fxa7Dg100111*), five chalcone-flavanone isomerase-encoding genes (*Fxa7Bg202436*, *Fxa7Dg102232*, *Fxa7Ag202526*, *Fxa7Cg102357*, *Fxa7Dg101819*), and two dihy-droflavonol 4-reductase (DFR)-encoding genes (*Fxa2Ag103849*, *Fxa2Bg203601*), were also exhibited highest expression levels in Kaorino and lowest in 2012-W02 at all three fruit development stages. In addition, one 4-coumarate-CoA ligase (4CL)-encoding gene (*Fxa7Cg103039*) involved in the pathway of phenylpropanoid biosynthesis (ko00940) was also most expressed in Kaorino and lowest expressed in 2012-W02 at all three fruit development stages (**Table 3**; **Supplementary Dataset S7**). In contrast to these genes, the vinorine synthase (shikimate O-hydroxycinnamoyltransferase, HCT)-encoding gene (*Fxa4Cg202053*), annotated in pathways of phenylpropanoid biosynthesis (ko00940) and flavonoid biosynthesis (ko00941), showed lowest expression level in Kaorino and highest in 2012-W02 (**Table 3**; **Supplementary Dataset S7**).

**Table 3:** The 19 DEGs and 18 TFs related to fruit skin color of strawberry.

Transcript_id	Compared groups (Regulated)			Annotated gene	Annotated pathway
	2012-W02 -vs.- Kaorino	2012-W02 -vs.- Fenyu NO.1	Fenyu NO.1 -vs.- Kaorino		
Fxa1Bg201013	Up	Up	Up	flavanone 3-hydroxylase (*F3H*)	Flavonoid biosynthesis (ko00941)
Fxa1Ag101086	Up	Up	Up		
Fxa1Cg101029	Up	Up	Up		
Fxa1Dg200969	Up	Up	Up		
Fxa7Ag200133	Up	Up	Up	chalcone synthase 1-like (*CHS*)	
Fxa7Bg200132	Up	Up	Up		
Fxa7Cg100092	Up	Up	Up		
Fxa7Dg100111	Up	Up	Up		
Fxa7Bg202436	Up	Up	Up	Chalcone-flavanone isomerase (*CHI*)	
Fxa7Dg102232	Up	Up	Up		
Fxa7Ag202526	Up	Up	Up		
Fxa7Cg102357	Up	Up	Up		
Fxa7Dg101819	Up	Up	Up		
Fxa2Ag103849	Up	Up	Up	dihydroflavonol 4-reductase (*DFR*)	
Fxa2Bg203601	Up	Up	Up		
Fxa7Cg103039	Up	Up	Up	4-coumarate–CoA ligase (*4CL*)	Phenylpropanoid biosynthesis (ko00940)
Fxa7Ag203300	Up	Up	Up	anthocyanidin 3-O-glucosyltransferase (*BZ1*)	Anthocyanin biosynthesis (ko00942)
Fxa7Dg102859	Up	Up	Up		
Fxa4Cg202053	Down	Down	Down	vinorine synthase (*HCT*)	Phenylpropanoid biosynthesis (ko00940); Flavonoid biosynthesis (ko00941)
Fxa6Cg101113	Down	Down	Down	transcription factor MYB -transcription factor AS1-like	–
Fxa1Bg201911	Up	Up	–	R2R3-MYB transcription factor MYB10-1	Circadian rhythm - plant (ko04712)
Fxa2Bg201632	Up	Up	Up	transcription factor bHLH62	Plant-pathogen interaction (ko04626)
Fxa2Dg202804	Up	Up	–	transcription factor bHLH74	
Fxa3Bg203691	Down	Down	Down	transcription factor bHLH122-like	
Fxa3Cg103622	Down	Down	Down		
Fxa4Bg103194	Down	Down	–	transcription factor bHLH79 isoform X3	
Fxa6Ag102265	Down	Down	Down	transcription factor bHLH130	
Fxa6Dg101934	Up	Up	–	transcription factor bHLH48	
Fxa1Ag102202	Up	Up	–	WD-40 domain protein 7	Ubiquitin mediated proteolysis (ko04120)
Fxa2Dg200558	Up	–	–		
Fxa3Cg100273	–	Up	–		
Fxa1Bg202086	–	Up	–		
Fxa1Cg102222	Up	Up	–		
Fxa1Dg202079	Up	Up	–		
Fxa4Ag103297	Up	–	–		
Fxa3Cg100366	Up	–	–	WD40 repeat-containing subunit B1	–
Fxa1Cg102117	Down	Down	Down	WRKY transcription factor 13	Plant-pathogen interaction (ko04626)

- means no significant difference, – means no annotated information.

### 3.6 Transcription factors involved in anthocyanin biosynthesis

**Figure 5:**
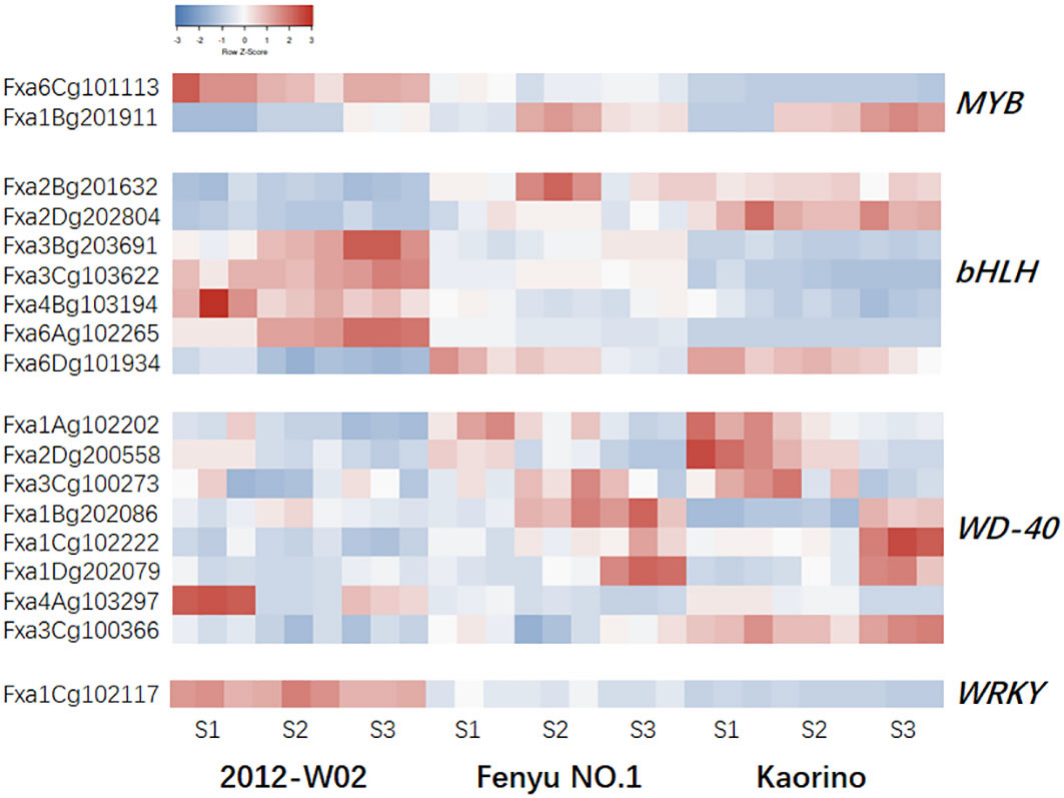
The expression pattern of TFs in Kaorino, 2012-W02 and Fenyu NO.1 at stages of S1, S2 and S3. The red color means high accumulated, and the blue means low accumulated of metabolites. Each column represents a sample; each sample has three replicates; each row represents a gene.

The skin color of strawberry fruits is influenced not only by genes that directly catalyzing anthocyanin biosynthesis but also by TFs. A total of 559 differentially expressed TFs were identified in strawberries of different skin colors at different stages, including members of the *MYB*, *bHLH*, and *WRKY* families (**Supplementary Dataset S8**). To investigate the TFs essential for anthocyanin biosynthesis in strawberry skin color formation, we focus on the four TF families, *MYB*, *bHLH*, *WD40* and *WRKY*. A total of 18 TFs were identified and differentially expressed among three accession samples at different developmental stages (**Figure 5**; **Table 3**). For the MYB family of TFs, the gene *Fxa6Cg101113*, which encodes an AS1-like protein was up-regulated in white skin accession 2012-W02 and down-regulated in red skin accession Kaorino and pink skin accession Fenyu NO.1 at three stages (**Figure 5**; **Table 3**). In addition, the *R2R3-MYB* member *MYB10-1* (*Fxa1Bg201911*) was significantly downregulated in white skin accession 2012-W02 (**Figure 5**; **Table 3**). It showed lower expression levels at the S1 and S2 stages compared to Fenyu NO.1, and lower expression at the S2 stage when compared to Kaorino (**Table 3**; **Supplementary Dataset S7**). Seven *bHLH* family members were found to be differentially expressed between the red skin accession Kaorino and the white skin accession 2012-W02 at all three developmenqtal stages (**Figure 5**; **Table 3**). Among them, three *bHLHs* (*Fxa2Bg201632*, *Fxa2Dg202804* and *Fxa6Dg101934*) were upregulated in Kaorino compared to 2012-W02, while four TFs (*Fxa3Bg203691*, *Fxa3Cg103622*, *Fxa4Bg103194* and *Fxa6Ag102265*) were downregulated in Kaorino compared to 2012-W02. The *Fxa2Bg201632* gene, which encodes bHLH62, was upregulated in the pink skin accession Fenyu NO.1 at three developmental stages compared with the white skin accession 2012-W02 (**Figure 5**; **Table 3**). In addition, the *Fxa6Ag102265* gene encoding bHLH130 was downregulated in the Kaorino at three developmental stages compared with Fenyu NO.1 (**Table 3**; **Supplementary Dataset S7**). For the *WD40* family of TFs, the eight genes were expressed at lower levels in 2012-W02 than in Kaorino and Fenyu NO.1, three of them (*Fxa1Bg202086*, *Fxa1Cg102222* and *Fxa1Dg202079*) were downregulated in 2012-W02 at S3 stage as compared to Kaorino and Fenyu NO.1 (**Table 3**; **Supplementary Dataset S7**). In addition, the gene *Fxa1Cg102117*, annotated as *WRKY13*, was found to be differentially expressed in all comparison groups. Its expression decreased with increasing skin color, with the highest level observed in 2012-W02 and the lowest level in Kaorino (**Table 3**; **Supplementary Dataset S7**).

### 3.7 Conjoint analysis of DRMs and DEGs involved in anthocyanin biosynthesis

Numerous studies have shown that strawberry fruit color is mainly controlled by anthocyanin biosynthesis. In this study, pathways related to anthocyanin biosynthesis, including phenylpropanoid biosynthesis (ko00940) (https://www.kegg.jp/pathway/map00940), isoflavonoid biosynthesis (ko00943) (https://www.kegg.jp/pathway/map00943) and flavone and flavonol biosynthesis (ko00944) (https://www.kegg.jp/pathway/map00944) were commonly enriched in DRMs and DEGs.

**Figure 6:**
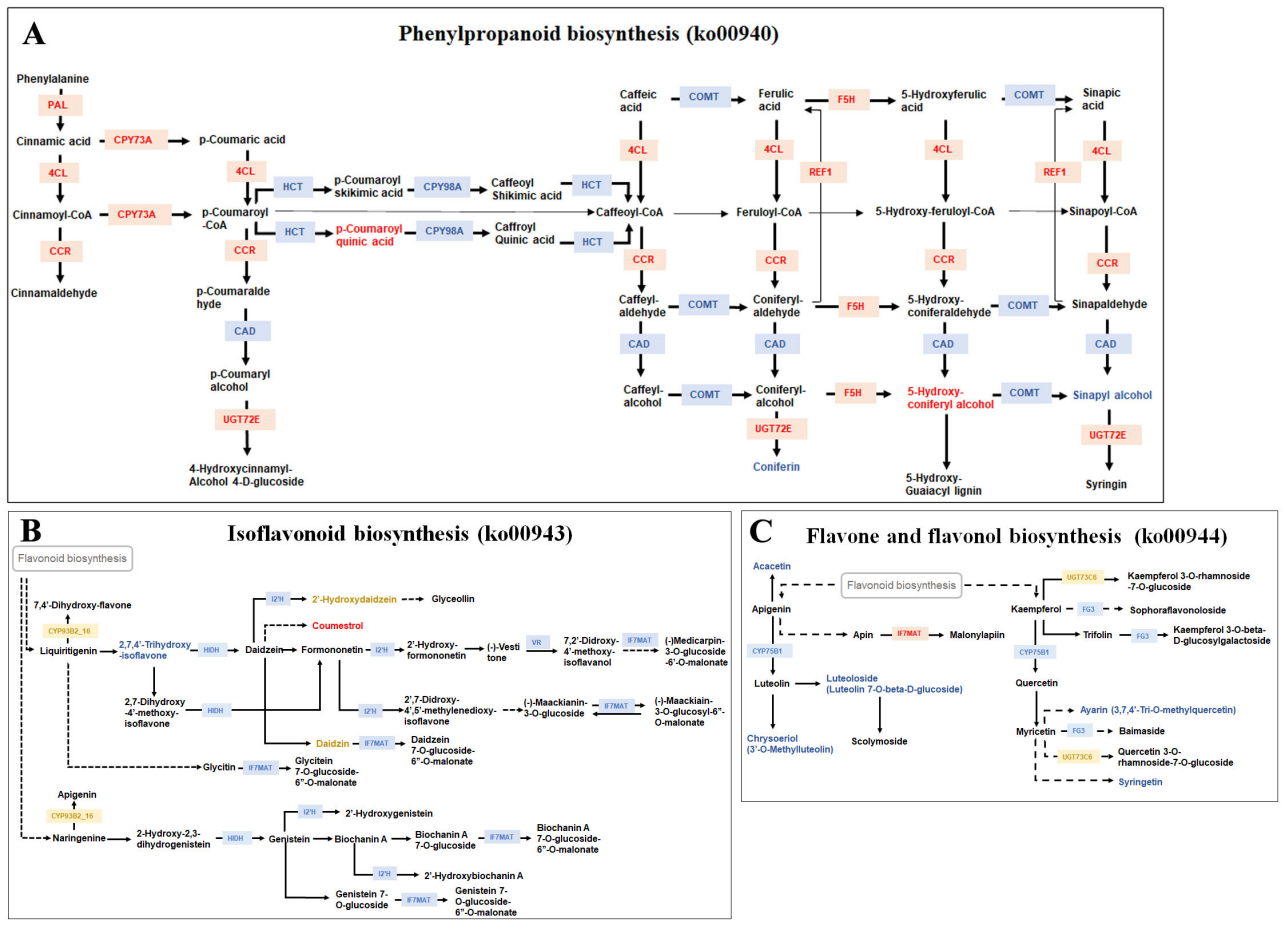
The commonly enriched KEGG pathways by DEGs and DRMs. **(A)** The phenylpropanoid biosynthesis pathway (origin from: https://www.kegg.jp/pathway/map00940). **(B)** The isoflavonoid biosynthesis pathway (origin from: https://www.kegg.jp/pathway/map00943). **(C)** The flavone and flavonol biosynthesis pathway (origin from: https://www.kegg.jp/pathway/map00944). The red text in each pathway is the DEGs or DRMs most expressed in red skin accession Kaorino compared with white skin accession 2012-W02 and pink skin accession Fenyu NO.1, the blue text is the DEGs or DRMs lowest expressed in red skin accession Kaorino compared with other two accessions. The yellow text is the DEGs or DRMs most expressed in pink skin variety Fenyu NO.1 compared with other two accessions.

In the phenylpropanoid biosynthesis pathway (ko00940), four metabolites, p-coumaroyl quinic acid, 5-hydroxyconiferyl alcohol, coniferin and sinapyl alcohol, and 21 genes, *PAL* (*Fxa6Dg101303*, *Fxa6Cg101365* and *Fxa6Ag101540*), *4CL* (*Fxa7Cg103039*), *HCT* (*Fxa4Cg202053*), *CAD* (*Fxa2Dg203268*), *CCR* (*Fxa7Ag200183*), *F5H* (*Fxa1Ag101125*), *REF1* (*Fxa1Ag100449*), *UGT72E* (*Fxa1Bg201201*, *Fxa1Dg201152*), *CYP73A* (*Fxa3Bg203470*, *Fxa4Bg100519*, *Fxa3Ag103799*, *Fxa3Dg203252*, *Fxa3Cg103403*), *CYP98A* (*Fxa1Dg202656*) and *COMT* (*Fxa7Bg203060*, *Fxa7Dg102804*, *Fxa7Cg102967* and *Fxa7Ag203226*), were differentially accumulated or expressed in the three accessions with different skin color. The levels of p-coumaroyl quinic acid and 5-hydroxyconiferyl alcohol were found to be higher in the red skin accession Kaorino compared to 2012-W02 and Fenyu NO.1. Consistently, the expression level of *F5H*, an upstream gene involved in 5-hydroxyconi coumaroyl feryl alcohol biosynthesis, was higher in the red skin accession Kaorino than in 2012-W02 and Fenyu NO.1. In addition, the metabolites of coniferin and sinapyl alcohol were up-regulated in the white skin accession 2012-W02 compared to red skin accession Kaorino and pink skin accession Fenyu NO.1 (**Figure 6**; **Supplementary Dataset S9**). Correspondingly, five upstream genes associated with these two metabolites, including four *COMT* genes and the *CAD* gene, were also found to be upregulated in 2012-W02 when compared to Kaorino and Fenyu NO.1. The *PAL* genes showed consistent upregulation in red skin accession Kaorino at all the three developmental stages compared to Fenyu NO.1 and 2012-W02. Additionally, these genes were also found to be upregulated in pink skin accession Fenyu NO.1 at the S2 and S3 stages when compared with 2012-W02. Similarly, gene *4CL* showed the highest expression level in red skin accession Kaorino, while the lowest expression level in 2012-W02 across all three developmental stages (**Figure 6**; **Supplementary Dataset S9**). *CCR*, *CPY73A*, and *UGT72E* genes were also highly expressed in red skin accession Kaorino. The expression of the *REF1* gene was significantly higher in Kaorino and Fenyu NO.1 compared to 2012-W02, with the highest level observed in Fenyu NO.1. In contrast, the expression levels of *HCT* and *CYP98A* were decreased as the skin color deepened in all three developmental stages, which exhibited a highest in white skin accession 2012-W02.

In the isoflavonoid biosynthesis pathway (ko00943), four DRMs, coumestrol, 2’-Hydroxydaidzein, 2,7,4’-Trihydroxyisoflavanone and daidzin, and six DEGs, *I2’H* (*Fxa2Dg202884*), *CYP93B2_16* (*Fxa5Ag203364*), *HIDH* (*Fxa4Ag101504*, *Fxa2Ag101983*), *IF7MAT* (*Fxa7Ag200152*), and *VR* (*Fxa7Cg101124*), were identified (**Figure 6**; **Supplementary Dataset S9**). Among them, the content of coumestrol was upregulated in the red skin accession Kaorino compared to Fenyu NO.1 at both S2 and S3 stages and compared to 2012-W02 at S2 stage. Conversely, 2,7,4’-trihydroxyisoflavanone was downregulated in the red skin accession Kaorino compared to 2012-W02 at all three stages. The metabolites 2’-hydroxydaidzein and daidzin were more abundant in the pink-skinned accession Fenyu NO.1 than in Kaorino and 2012-W02. Similarly, the expression level of the *CYP93B2_16* was also highest in the pink-skinned accession Fenyu NO.1 across the three accessions (**Figure 6**; **Supplementary Dataset S9**). Interestingly, the expression levels of all the DEGs in this pathway were found to be up-regulated in the white skin accession 2012-W02 compared to red and pink skin accessions (**Figure 6**; **Supplementary Dataset S9**).

A total of five DRMs, syringetin, 3,7,4’-Tri-O-methylquercetin, chrysoeriol, acacetin, luteolin 7-O-beta-D-glucoside, and nine DEGs, *CYP75B1* (*Fxa5Dg201116*), *FG3* (*Fxa5Dg201948*), *UGT73C6* (*Fxa6Bg101474*) and *IF7MAT* (*Fxa6Bg104065*, *Fxa6Bg104066*, *Fxa6Ag104642*, *Fxa7Ag200152*, *Fxa6Cg103966*, and *Fxa6Ag104436*), were identified in the flavone and flavonol biosynthesis pathway (ko00944) (**Figure 6**; **Supplementary Dataset S9**). All the five metabolites showed the highest accumulation in the white skin accession 2012-W02 and the lowest accumulation in the red skin accession Kaorino). Interestingly, the upstream gene of these DRMs, *CYP75B1*, which encodes flavonoid 3’-monooxygenase, was also upregulated in 2012-W02 compared to Kaorino and Fenyu NO.1 (**Figure 6**; **Supplementary Dataset S9**). Similarly, the *FG3* gene, which encodes flavonol-3-O-glucoside/galactoside glucosyltransferase, showed the highest expression in white skin accession 2012-W02 compared to the other two accessions (**Figure 6**; **Supplementary Dataset S9**). The gene *UGT73C6*, which encodes flavonol-3-O-L-rhamnoside-7-O-glucosyltransferase, showed the highest expression level in pink skin accession Fenyu NO.1 at the S3 stage compared to 2012-W02 and Kaorino. The six *IF7MAT* genes, encoding isoflavone 7-O-glucoside-6’’-O-malonyltransferase showed higher expression levels in the red skin accession Kaorino than in pink skin accession Fenyu NO.1 and white skin accession 2012-W02.

### 3.8 qRT-PCR validation

**Figure 7:**
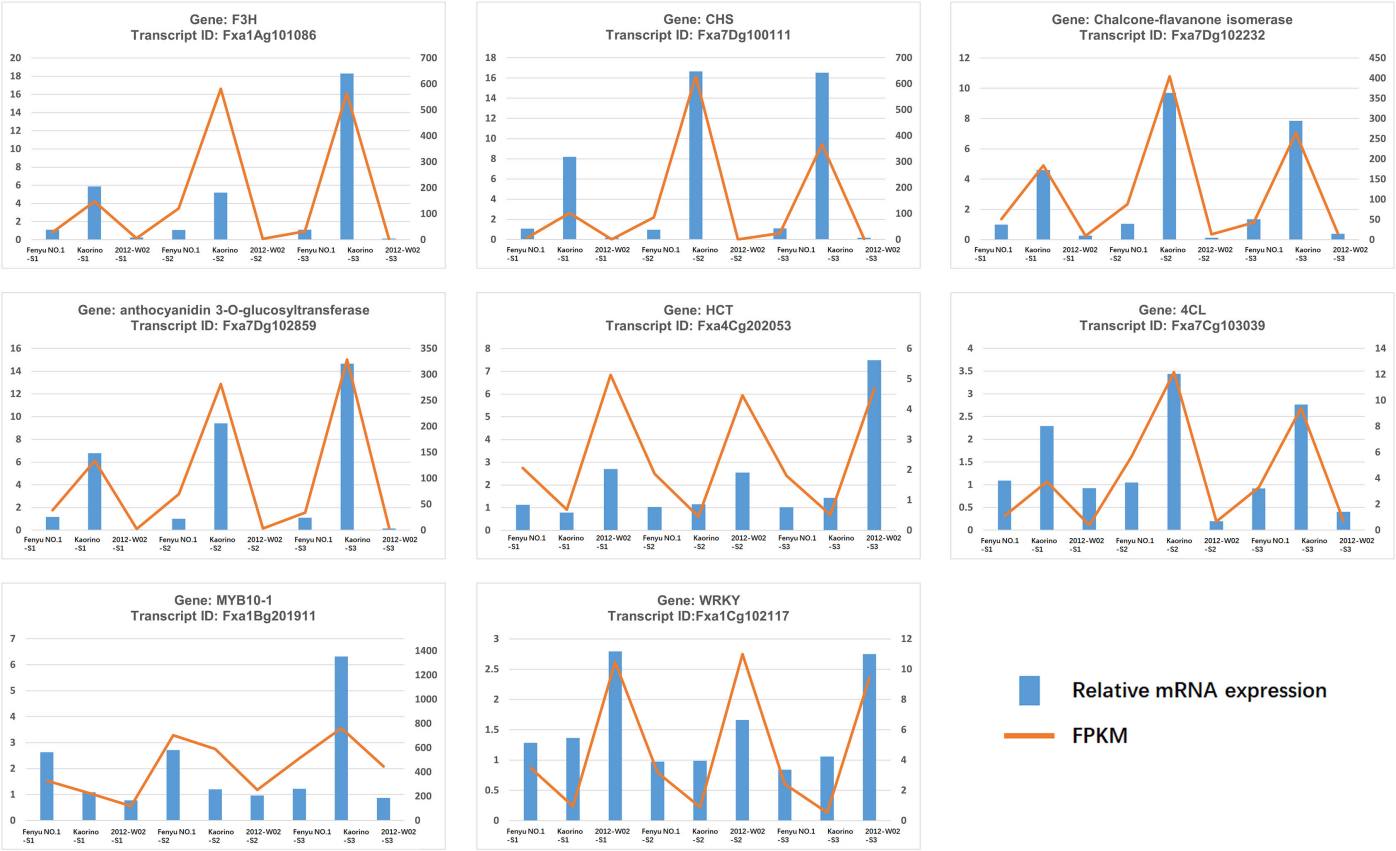
qRT-PCR validation of 8 DEGs. The x-axis represents the tissue samples while the left y-axis represents the relative mRNA expression and right y-axis represents the FPKM value of each gene.

To validate the RNA-Seq results, eight DEGs associated with anthocyanin biosynthesis were selected for qRT-PCR validation. The transcriptional levels of these genes determined by qRT-PCR analysis showed a similar trend to that observed in the RNA-Seq data (**Figure 7**), indicating the high reliability of the RNA-Seq data.

## 4 Discussion

Fruit skin color is a highly variable trait controlled by relatively complex genetic mechanisms. Previous studies have shown that fruit color is caused by plant pigments, including lycopene, anthocyanins and carotenoids, and that the formation of strawberry fruit color is mainly due to anthocyanin accumulation (Lin et al., 2023; Yue et al., 2023). Anthocyanin biosynthesis in fruits has become an interesting and useful area of research due to the need to better understand its mechanism and to develop fruit cultivars with relatively high anthocyanin content. Carolina and his colleagues studied the mechanism of anthocyanin synthesis and transcription factor regulation in strawberry fruit ripening and development using red fruits (Parra-Palma et al., 2020). They found that there was a correlation between redness and total anthocyanin content, and the expression levels of *FaF3H* and *FaFLS* were positively correlated with total flavonoid content at the early stage of ripening (Parra-Palma et al., 2020). In addition, Duan et al. reported that the expression levels of anthocyanin biosynthesis-related genes, including *FpCHS, FpDFR, FpANS* and *FpUFGT*, as well as the regulatory gene *MYB10*, were significantly upregulated in red strawberry fruits compared to white strawberry fruits (Duan et al., 2017). Bulk sequencing analysis of strawberry revealed that the *RAP* gene, which encodes glutathione S-transferase, can bind to anthocyanin and promote anthocyanin transport from the cytosol to the vacuoles (Luo et al., 2018). *RAP* has been shown to be important for fruit coloration in strawberry plants (Gao et al., 2020). Although these studies have identified genes related to strawberry fruit coloration and elucidated the underlying mechanism of strawberry fruit coloration, most of them have used red strawberry fruits as a material. However, our current understanding of pigmentation in strawberry plants is still limited. In this study, we selected the red fruit skin accession Kaorino, the white fruit skin accession 2012-W02, and their F1 offspring Fenyu NO.1, which exhibits pink-skinned phenotype, as experimental materials to investigate the molecular mechanisms underlying the differential regulation of strawberry fruit skin coloration through transcriptome and metabolome analyses.

Flavonoids are the most abundant secondary metabolites in plants, which not only provide protection against UV light, pests and diseases, but also play a crucial role in tissue coloration (Dwibedi et al., 2022). Anthocyanins, flavanones, flavanonols, flavonols, and flavanols are the five major subclasses of flavonoids (Han et al., 2023). Anthocyanins are the most prominent class of flavonoids and are responsible for the coloration of flowers, fruits, seeds and leaves (Owens et al., 2008). Anthocyanins are synthesized and accumulate in the pericarp during the ripening stage (Ferreira et al., 2018). At the white stage of strawberry fruit development, the total anthocyanin content decreases, and more total anthocyanins are found in ripe strawberry fruits (Hossain et al., 2018). In this study, the accumulation of a specific anthocyanin metabolite (peonidin 3-O-glucoside) showed variation among white skin, red skin and pink skin strawberry accessions (**Figure 2**). In addition, the levels of anthocyanin-related metabolites, such as p-Coumaroyl quinic acid, 5-Hydroxyconiferyl alcohol, syringetin, and 2,7,4’-trihydroxy-isoflavone, were also varied among these three accessions (**Figure 2**). Metabolomic analysis revealed a higher number of DRMs between the white skin and red/pink samples than between the red and pink samples at all three developmental stages (**Figure 3A**). These DRMs were significantly enriched in glyoxylate and dicarboxylate metabolism, amino acid metabolism, biosynthesis of phenylpropanoids, flavone and flavonol biosynthesis and isoflavonoid biosynthesis (**Supplementary Figure S2**). These results indicate that the accumulation of different subclasses of flavonoids plays a significant role in determining the skin color of strawberry plants.

Anthocyanins are the major pigments responsible for the coloration strawberry fruit (Zhao et al., 2021). The anthocyanin content of strawberry fruit is mainly due to the accumulation of cyanidin (dark red color) and pelargonidin (light red color), with the pelargonidin content shown to be greater than that of cyanidin in fruit (Härtl et al., 2017; Zhang et al., 2020). It has been confirmed that anthocyanins are derived from the flavonoid biosynthetic pathway (Schijlen et al., 2004). Genes related to the flavonoid biosynthesis pathway, including *CHS*, *CHI*, *F3H*, *DFR*, *ANS* and *3-GT* (3-glycosyltransferase), have been shown to determine the color of strawberry fruits (Salvatierra et al., 2010). The enzyme F3H catalyzes flavanones to dihydroflavonols. In subsequent biosynthetic steps, the *DFR* reduces dihydroflavonols to leucoanthocyanins, which are further converted to anthocyanidins by LDOX/ANS (Hichri et al., 2011). In the present study, the RNA-seq data showed that the genes of *CHS*, *CHI*, *F3H* and *DFR* were all up-regulated in red skin accession Kaorino, while down-regulated in white skin accession 2012-W02 at all three fruit development stages (**Table 3**). The gene *BZ1*, encoding anthocyanidin-3-O-glucosyltransferase, catalyzes the formation of the first stable intermediate in the anthocyanin pathway (Griesser et al., 2008). There is almost no expression of *BZ1* in green strawberry fruits, while the expression of this gene increases dramatically in both turning and ripe red fruits (Griesser et al., 2008). Consistently, two *BZ1* genes were found to be significantly up-regulated in the red skin accession Kaorino compared to the white-skinned accession 2012-W02 (**Table 3**). Similarly, the *4CL* genes encoding the enzyme 4-coumarate: CoA ligase, in the phenylpropanoid biosynthesis pathway, were also up-regulated in Kaorino and down-regulated in 2012-W02 at the three fruit development stages. The *4CL* gene activates cinnamic acid and its hydroxylated derivatives by forming the corresponding CoA thioesters (Kumar and Ellis, 2003). In sweet cherry (*Prunus avium* L.), the expression level of *4CL* was found to be higher in the red fruit than in the yellow fruit (Wei et al., 2015). The *HCT* gene, which encodes hydroxycinnamoyl:CoA transferase, competes with *4CL* by catalyzing the conversion of 4-coumaryl-CoA to 4-coumaryl-shikimate and subsequently promoting the production of the S-P-G type of lignin through the downstream regulatory enzymes (Liu et al., 2018). The expression levels of *HCT* genes were lower in red skin peanut than in pink skin peanut (Ahmad et al., 2023). In consistent with previous study, the expression levels of the *HCT* genes identified in this study were found to be downregulated in red skin accession Kaorino and upregulated in white skin accession 2012-W02 at all three fruit development stages (**Table 3**). We suggest that the downregulation of *HCT* genes provides an alternative mechanism to promote anthocyanin accumulation by disrupting of lignin pathways, thereby influencing the coloration of strawberry fruit skin.

Anthocyanin accumulation is tightly regulated by transcription factors (TFs), including members of the MYB, bHLH, WD40 and WRKY protein families (Qi et al., 2011). These three TFs can regulate the structural genes involved in the anthocyanin synthesis pathway and can play a regulatory role in anthocyanin synthesis by forming the MYB-bHLH-WD40 (MBW) ternary complex (Xu et al., 2015). *MYB10* has been shown to play an essential role in the anthocyanin accumulation and distribution in strawberry (Castillejo and Waurich, 2020). Based on the analysis of transcriptome data and the detection of SNP mutation sites, Hawkins et al. speculated that a single amino acid mutation in the MYB10 protein was the main cause of anthocyanin deficiency in white strawberry fruits (Hawkins et al., 2016). In addition, the expression of *MYB10* was higher in red strawberry fruits than in white strawberry fruits (Zhang et al., 2015). Wang et al. also reported that overexpression of *MYB10* in white strawberry fruit significantly increased anthocyanin content in transgenic plants, after which the white fruit turned to red, while in *MYB10*-silenced plants, anthocyanin accumulation was significantly inhibited, resulting in a white-fruit phenotype (Lin-Wang et al., 2014). In this study, the *MYB10* gene was significantly downregulated in the white fruit skin accession 2012-W02 (**Table 3**), thereby suggesting its role as a regulatory gene in strawberry fruit skin coloration. A previous study showed that changes in the expression of *bHLH* and *WD40* do not directly affect anthocyanin accumulation, but rather regulate anthocyanin biosynthesis through the formation of MYB-bHLH-WD40 (MBW) (Li, 2014; Schaart et al., 2013). The expression level of *bHLH* was significantly up-regulated in white strawberry fruits compared to red strawberry fruits, while the transcription level of the *WD40* gene showed no significant change between the two accession phenotypes (Härtl et al., 2017). However, it was also found that the silencing of *bHLH33* had no significant effect on the key enzyme-encoding genes related to the anthocyanin synthesis pathway or the anthocyanin content (Lin-Wang et al., 2014). In our study, the genes annotated in *bHLH* and *WD40* were also detected as DEGs, and some of them were both up-regulated or down-regulated in red skin accession Kaorino. Therefore, the relationships between these two genes and anthocyanin accumulation are worthy of further study. Studies have shown that the WRKY protein TTG2 in *Arabidopsis* can promote anthocyanin accumulation by regulating the expression of the *TT12* and *TT13* genes on the vacuolar membrane (Duan et al., 2018; Gonzalez et al., 2016). Furthermore, it has been demonstrated that overexpression of *WRKY41* in *Brassica* significantly reduces anthocyanin content (Duan et al., 2018). Similarly, our results showed that the expression level of *WRKY13* (*Fxa1Cg102117*) was significantly higher in white skin accession 2012-W02 compared to red skin accession Kaorino. Although the effect of WRKY protein on anthocyanin accumulation has been reported, the mechanism by which *WRKYs* regulate anthocyanin metabolism is still unclear, and the relationship between *WRKYs* and plant coloration needs to be further investigated.

Anthocyanins are synthesized from three molecules of malonyl CoA derived from fatty acid metabolism and one of p-coumaroyl CoA synthesized from phenylalanine via the phenylpropanoid pathway (Kapoor and Sharma, 2023). The phenylpropanoid pathway is initiated by *PAL*, *C4H* and *4CL*, which give rise to cinnamate, p-coumarate and p-coumaroyl coA, respectively, and serve as the basis for all subsequent branches and metabolites (Yadav et al., 2020). The phenylpropanoid biosynthesis pathway (ko00940) was significantly enriched by DEGs and DRMs in the present study. The p-Coumaroyl quinic acid is the precursor of chlorogenic acid which is an important secondary metabolite in the phenylpropanoid pathway and is correlated with anthocyanins (Chen et al., 2020; Liao et al., 2021; Singh et al., 2018; Su et al., 2022). In this study, p-coumaroyl quinic acid was found to be up-regulated in the red-skinned accession Kaorino compared to white skin accession 2012-W02 (**Figure 6**; **Supplementary Dataset S9**). The synthesis of coniferyl and sinapyl alcohols requires the activity of O-methyltransferases (OMTs), and COMT deficient can result in more 5-hydroxyconiferyl alcohol in alfalfa (Marita et al., 2003). Corresponding to this expression model, the 5-hydroxyconiferyl alcohol was found to be upregulated in red skin Kaorino and the coniferin, sinapyl alcohol downregulated in Kaorino compared to 2012-W02 in this study. In addition, the genes encoding COMT were also downregulated in Kaorino and upregulated in 2012-W02. The *CCR* and *CAD* were the key enzymes for lignin formation, and the silencing of two *CAD* genes in *Nicotiana attenuata* resulted in red-pigmented stems, reflecting blocked lignification (Ackah et al., 2022; Wang et al., 2014; Kaur et al., 2012). In addition, the lignin formation related gene, such as *FaPRX27*, is also associated with strawberry fruit color during strawberry fruit ripening (Ring et al., 2013). In this study, the expression level of *CAD* gene was upregulated in the white-skinned accession 2012-W02, which is consistent with previous studies and suggests that the upregulation of the *CAD* gene diverts the flux from anthocyanins to lignin and subsequently affects the color of strawberry fruit skin.

In plants, the flavonoid pathway is the upstream pathway of isoflavonoid biosynthesis, so the regulatory pathway of anthocyanins also depends on the inhibition of isoflavonoid synthesis (Ahmad et al., 2023). The isoflavanoids are produced by 2-hydroxyisoflavanone synthase (IFS, CPY93C) and 2-hydroxyisoflavanone dehydratase (HIDH) enzymes using naringenin and/or liquiritigenin and then undergo several tailoring steps, such as glycosylation, methylation, and hydroxylation (Wang et al., 2015). In this study, the *I2’H*, *HIDH*, and *VR* genes, which encode enzymes involved in isoflavonoid biosynthesis, were significantly downregulated in red skin Kaorino and pink skin Fenyu NO.1 accessions compared with the white-skinned accession 2012-W02. Correspondingly, the relative levels of metabolites catalyzed by these DEGs, such as 2,7,4’-trihydroxyisoflavanone, were significantly lower in red skin accession Kaorino compared to white skin accession 2012-W02 (**Figure 6**; **Supplementary Dataset S9**). These findings suggest an apparent switch from the isoflavone biosynthetic pathway to anthocyanin biosynthesis in red skin strawberry.

The flavone and flavonol biosynthesis pathway (ko00944) is one of the branches of flavonoid biosynthesis. In this study, four DEGs (*CYP75B1*, *FG3*, *UGT73C6* and *IF7MAT*) and five DRMs (syringetin, ayarin, chrysoeriol, acacetin, and luteoloside) were identified in the flavone and flavonol biosynthesis pathway among strawberry accessions with different fruit skin colors (**Figure 6**; **Supplementary Dataset S9**). The DEGs and DRMs, except for the *IF7MAT* gene, were all up-regulated in the white fruit skin accession 2012-W02 compared to the red fruit skin accession Kaorino (**Figure 6**, **Supplementary Dataset S9**). Research has shown that white grape cultivars mainly synthesize the mono- and di-substituted B-ring derivatives kaempferol, quercetin and isorhamnetin (Flamini et al., 2013; Mattivi et al., 2006). Flavonoid 3’-monooxygenase (*CYP75B1*, *F3’H*) catalyzes the B-ring hydroxylation of flavonoids, including the 3’-hydroxylation of naringenin, dihydrokaempferol and kaempferol (Zhou et al., 2016). In this study, the *CYP75B1* gene, along with its downstream gene *FG3* and the metabolites astarin and syringetin, were up-regulated in white fruit skin when compared to red skin fruit (**Figure 6**; **Supplementary Dataset S9**), indicating a correlation between the DEGs and DRMs annotated in the flavone and flavonol biosynthesis pathway with strawberry skin color formation. In addition, the majority of the genes and metabolites identified in this pathway were up-regulated in the white fruit skin accession 2012-W02 compared to the red skin accession Kaorino, which likely resulted in restricted anthocyanin synthesis in the white fruit skin.

## 5 Conclusion

Fruit skin color is an important phenotypic trait and a major contributor to fruit quality and subsequent market value. In the present study, a combination of transcriptome and metabolome profiling approaches was used to gain deeper insights into the molecular mechanisms underlying skin color in strawberries, with the aim of elucidating the potential involvement of anthocyanin biosynthesis pathways in the formation of fruit skin color. We found that the formation of red fruit skin color is primarily attributed to pathways related to anthocyanin biosynthesis. The genes *BZ1, F3H, CHS, CHI, DFR, HCT*, *4CL, CYP75B1, FG3, HIDH, IF7MAT, I2’H, VR, PAL, CCR, F5H*, *REF1* and *UGT72E*, which play important roles in anthocyanin biosynthesis, flavonoid biosynthesis, isoflavonoid biosynthesis and phenylpropanoid biosynthesis, were identified as essential contributors to skin color in strawberry. In addition, the transcription factors *MYB10* and *WRKY13* were also identified to be differentially expressed among strawberry skins with different color. Furthermore, several metabolites in the flavonoid-related pathways, including luteoloside, chrysoeriol, ayarin, syringetin, 2,7,4’-trihydroxy-isoflavone, p-coumaroyl quinic acid and 5-hydroxyconiferyl alcohol, also showed a strong correlation with the coloration of strawberry fruit skin. In summary, the DEGs, DRMs and significantly enriched pathways identified in the present study provide an important theoretical basis for understanding the process of strawberry skin pigmentation.

## Data Availability

The datasets of transcriptome data generated during the current study are available in SAR (sequence read archive) of NCBI [Accession number: PRJNA987176].
