# Supplementary material for: Combined transcriptome and metabolome analysis revealed the molecular mechanisms of fruit skin coloration in pink strawberry
Source: Front Plant Sci. 2024 Oct 10;15:1486892. doi: 10.3389/fpls.2024.1486892 (PMC11499181; doi:10.3389/fpls.2024.1486892)
Supplement: Supplementary file 1 [file DataSheet1.docx]

Supplementary Material

## Supplementary Tables

**Table S1. The primers of 8 validated genes for qRT-PCR.**

| **Transcript_id** | **Primer name**  **(Sense/Anti-sense primer)** | **Nucleotide sequence** |
| --- | --- | --- |
| Fxa1Ag101086 | F | 5' ATCGTTTCCAGCCATTTA 3' |
|  | R | 5' CATTCTTGAACCTCCCATT 3' |
| Fxa7Cg103039 | F | 5' GCAGCTGTTGTTCCGCAAA 3' |
|  | R | 5' TGCATGCACGAAGTACACCT 3' |
| Fxa7Dg100111 | F | 5' AAATTCCAGCGCATGTGTGAC 3' |
|  | R | 5' CTTAATGGCCTTGACAGCGG 3' |
| Fxa7Dg102232 | F | 5' TTCAGAAGTAGTGTTGGTGGAT 3' |
|  | R | 5' CAGGAGCAGAAATAAGAGCA 3' |
| Fxa7Dg102859 | F | 5' AATCATCTTCGGAAACTTGG 3' |
|  | R | 5' GGAACTCATCTAGCTGTGGG 3' |
| Fxa4Cg202053 | F | 5' GTCCACGGAACCCAATAC 3' |
|  | R | 5' CGCAAACATCGTTACCCTC 3' |
| Fxa1Bg201911 | F | 5' GTCCACGGAACCCAATAC 3' |
|  | R | 5' CGCAAACATCGTTACCCTC 3' |
| Fxa1Cg102117 | F | 5' AGCCTTGAGTTCCTTACC 3' |
|  | R | 5' CACTTTCCTCCGAATAGC 3' |
| FaACTIN (reference) | F | 5' GGGCCAGAAAGATGCTTATGTCGG 3' |
|  | R | 5' GGGCAACACGAAGCTCATTGTAGAAG 3' |

**Table S2. The statistic of RNA-Seq on 27 samples.**

| **Samples** | **Clean reads** | **Mapped reads (%)** | **GC Content (%)** | **Q30 (%)** |
| --- | --- | --- | --- | --- |
| 2012-W02_S1-1 | 42,201,920 | 39,696,567 (94.06%) | 47.35% | 93.92% |
| 2012-W02_S1-2 | 42,711,454 | 40,640,286 (95.15%) | 47.36% | 94.09% |
| 2012-W02_S1-3 | 38,716,130 | 36,512,566 (94.31%) | 47.35% | 94.01% |
| 2012-W02_S2-1 | 41,714,640 | 39,639,275 (95.02%) | 46.94% | 93.39% |
| 2012-W02_S2-2 | 43,588,678 | 41,382,395 (94.94%) | 46.93% | 93.47% |
| 2012-W02_S2-3 | 41,481,928 | 39,335,711 (94.83%) | 46.85% | 93.46% |
| 2012-W02_S3-1 | 39,263,632 | 37,204,991 (94.76%) | 46.65% | 94.06% |
| 2012-W02_S3-2 | 49,159,520 | 46,613,901 (94.82%) | 46.51% | 93.37% |
| 2012-W02_S3-3 | 38,334,788 | 36,360,914 (94.85%) | 46.47% | 94.47% |
| Kaorino _S1-1 | 41,867,638 | 39,598,462 (94.58%) | 46.99% | 93.88% |
| Kaorino _S1-2 | 42,775,340 | 40,918,152 (95.66%) | 47.11% | 93.50% |
| Kaorino _S1-3 | 41,917,632 | 39,788,225 (94.92%) | 46.98% | 93.75% |
| Kaorino _S2-1 | 42,797,242 | 40,610,148 (94.89%) | 46.92% | 93.87% |
| Kaorino _S2-2 | 42,956,222 | 40,866,756 (95.14%) | 46.93% | 93.84% |
| Kaorino _S2-3 | 42,126,928 | 39,946,527 (94.82%) | 46.91% | 93.39% |
| Kaorino _S3-1 | 42,807,798 | 40,495,190 (94.60%) | 46.54% | 93.13% |
| Kaorino _S3-2 | 50,135,452 | 47,175,410 (94.10%) | 46.58% | 94.02% |
| Kaorino _S3-3 | 53,015,656 | 50,224,230 (94.73%) | 46.63% | 94.25% |
| Fenyu NO.1_S1-1 | 41,690,278 | 38,638,229 (92.68%) | 47.02% | 93.17% |
| Fenyu NO.1_S1-2 | 38,297,972 | 36,404,972 (95.06%) | 46.92% | 93.83% |
| Fenyu NO.1_S1-3 | 42,193,812 | 39,540,868 (93.71%) | 47.00% | 93.98% |
| Fenyu NO.1_S2-1 | 44,953,726 | 42,431,055 (94.39%) | 46.72% | 93.15% |
| Fenyu NO.1_S2-2 | 44,069,620 | 41,835,571 (94.93%) | 46.68% | 94.08% |
| Fenyu NO.1_S2-3 | 41,046,188 | 38,950,732 (94.89%) | 46.71% | 94.27% |
| Fenyu NO.1_S3-1 | 46,290,372 | 43,724,177 (94.46%) | 46.38% | 93.89% |
| Fenyu NO.1_S3-2 | 50,152,806 | 47,554,843 (94.82%) | 46.35% | 93.81% |
| Fenyu NO.1_S3-3 | 48,418,782 | 44,859,018 (92.65%) | 46.51% | 93.82% |

## Supplementary Figures

| **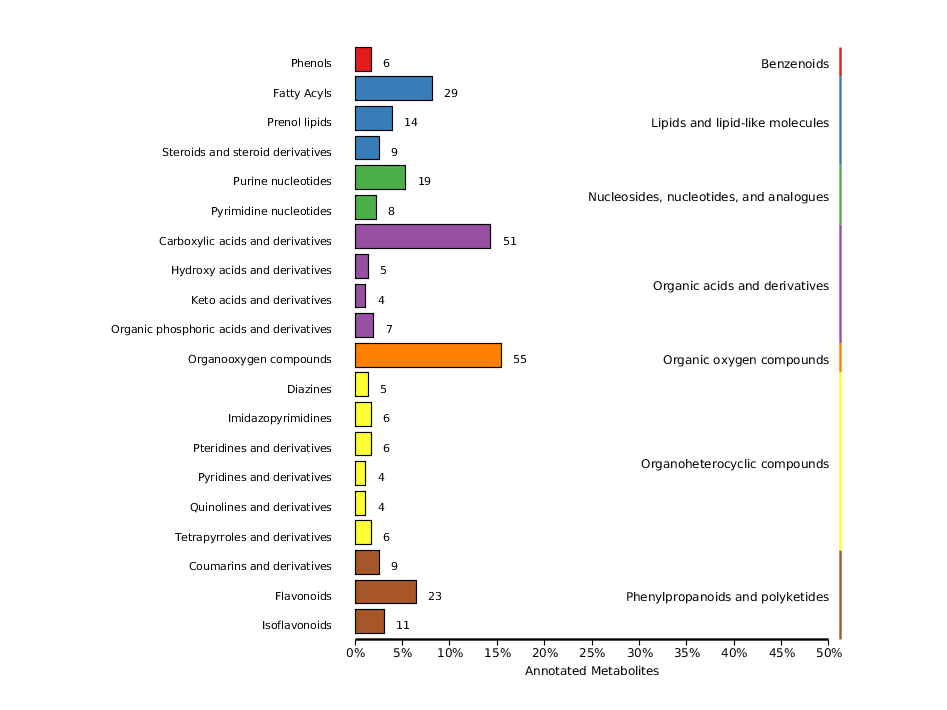**   1. The annotated result of metabolites in HMDB database. |
| --- |
| **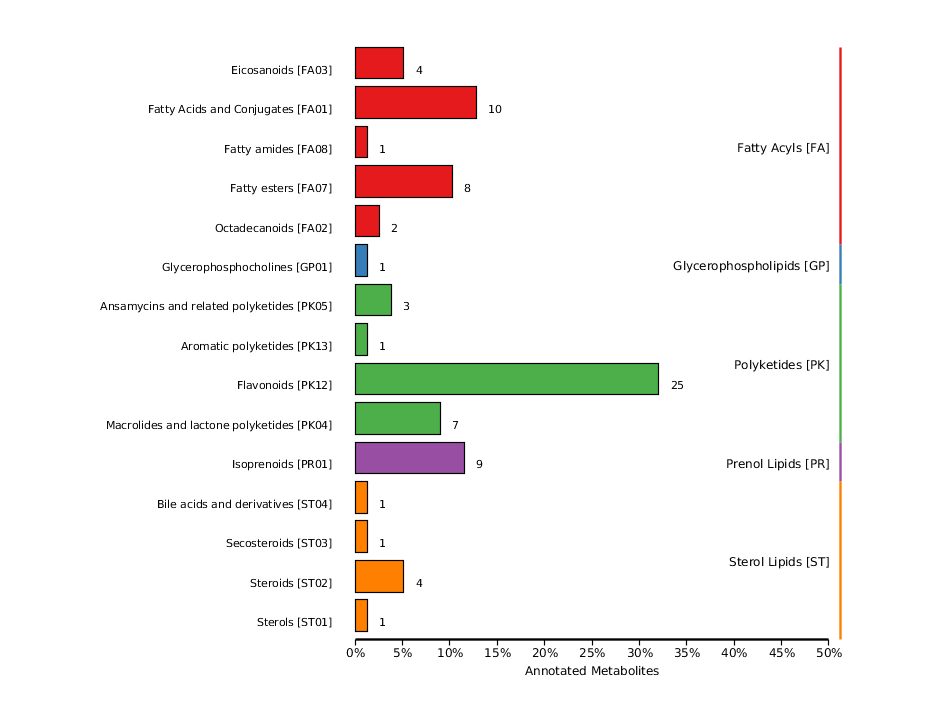**  B. The annotated result of metabolites in Lipidmaps database. |

**Supplementary Figure 1.** The Statistic of detected metabolites annotated in HMDB and Lipidmaps database.

| **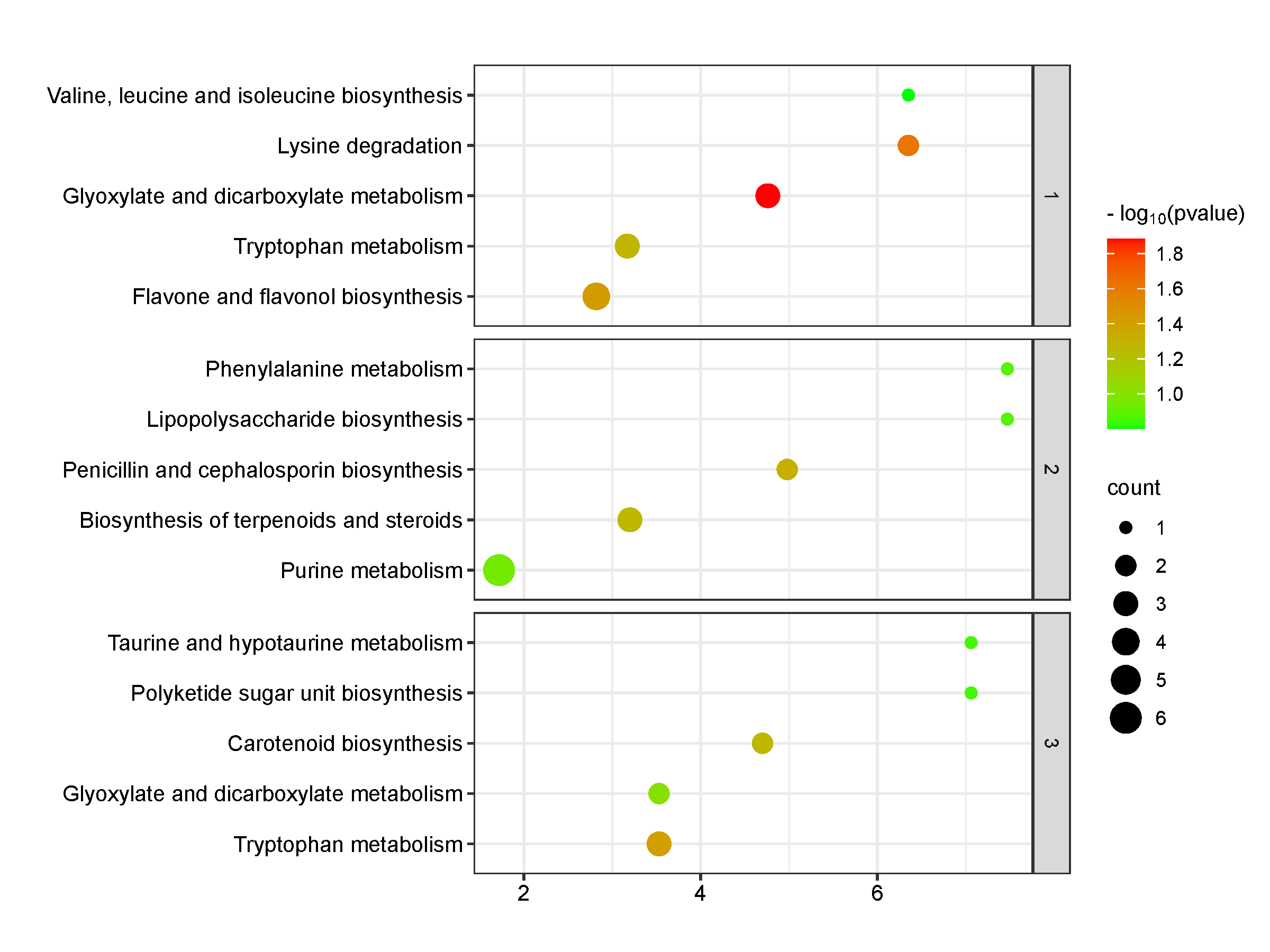**   1. The KEGG annotation of DEGs in compared group of 2012-W02 -vs.- Kaorino. |
| --- |
| **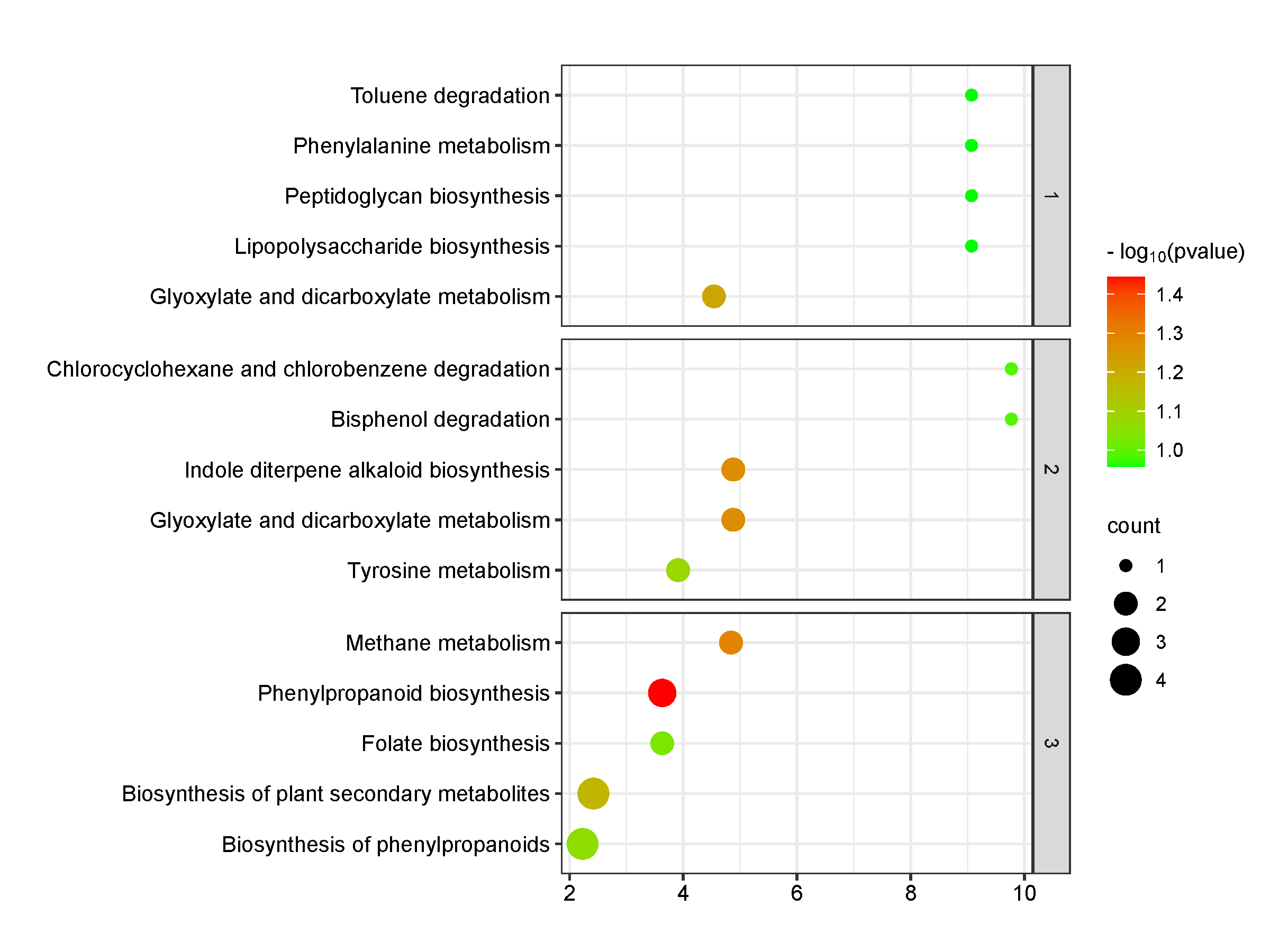**   1. The KEGG annotation of DEGs in compared group of 2012-W02 -vs.- Fenyu NO.1. |
| **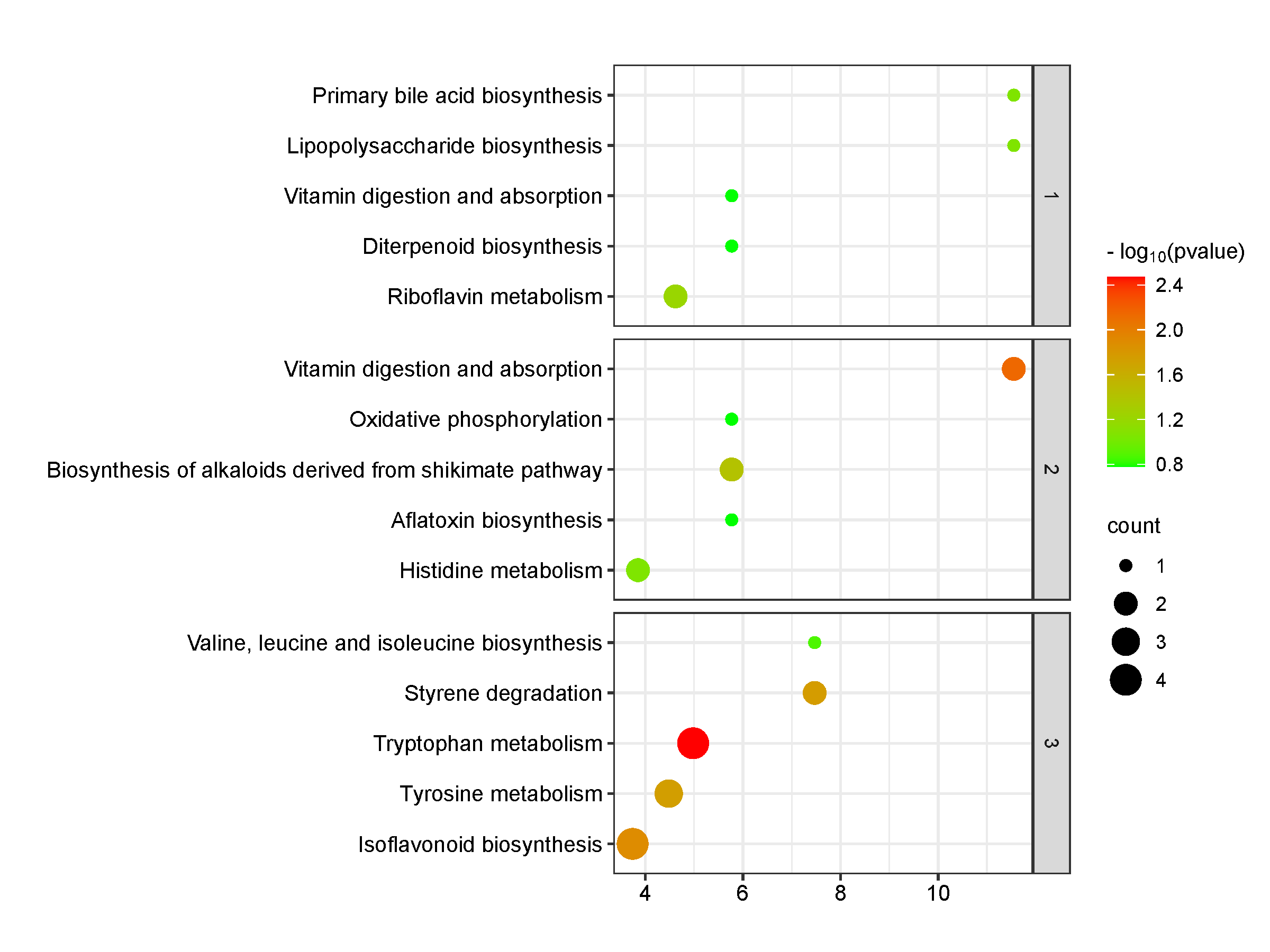**   1. The KEGG annotation of DEGs in compared group of Fenyu NO.1-vs.- Kaorino. |

**Supplementary Figure 2.** The KEGG annotation of DRMs in three compared groups at three stages of S1, S2 and S3 (The number of 1, 2, 3 in grey box in the right means the stages of S1, S2 and S3, respectively).
